# The Role of Glycans in Human Immunity—A Sweet Code

**DOI:** 10.3390/molecules30132678

**Published:** 2025-06-20

**Authors:** Igor Tvaroška

**Affiliations:** Institute of Chemistry, Slovak Academy of Sciences, 845 38 Bratislava, Slovakia; chemitsa@savba.sk

**Keywords:** glycans, glycosylation, carbohydrate determinants, glycan-binding proteins, leukocyte adhesion cascade, host-pathogen, self/nonself/altered nonself, cancer, glycan antibodies, glycan vaccines

## Abstract

Glycans on the surface of all immune cells are the product of diverse post-translational modifications (glycosylation) that affect almost all proteins and possess enormous structural heterogeneity. Their bioinformational content is decoded by glycan-binding proteins (lectins, GBPs), such as C-type lectins, including selectins, galectins, and Siglecs. Glycans located on the surface of immune cells are involved in many immunological processes through interactions with GBPs. Lectins recognize changes in the glycan epitopes; distinguish among host (self), microbial (non-self), and tumor (modified self) antigens; and consequently regulate immune responses. Understanding GBP–glycan interactions accelerates the development of glycan-targeted therapeutics in severe diseases, including inflammatory and autoimmune diseases and cancer. This review will discuss *N*- and *O*-glycosylations and glycosyltransferases involved in the biosynthesis of carbohydrate epitopes and address how interactions between glycan epitopes and GBPs are crucial in immune responses. The pivotal role of the glycan antigen tetrasaccharide sialyl Lewis x in mediating immune and tumor cell trafficking into the extravascular site will be discussed. Next, the role of glycans in modulating bacterial, fungal, viral, and parasitic infections and cancer will be surveyed. Finally, the role of glycosylation in antibodies and carbohydrate vaccines will be analyzed.

## 1. Introduction

Glycans are significant components of the gel-like layer (the glycocalyx) covering the surface of most eukaryotic cells. The structure of glycans is diverse, ranging from the simplest monosaccharides (e.g., *N*-acetylglucosamine or galactopyranose) to complex *N*- and *O*-glycans. The biosynthesis of glycans (glycosylation) occurs in the endoplasmic reticulum (ER) and Golgi apparatus by glycoside hydrolases (GHs) and glycosyltransferases (GTs). Different types of glycosylation exist, and the two most frequently occurring are *N*- and *O*-glycosylation. Glycosylation is a contingent cellular process resulting from non-template-directed secondary gene events. This complex secondary gene event, a post-translational modification (PTM), results in a heterogeneous mixture of structurally similar but distinct glycan motifs linked to a single protein, known as glycoforms. It seems that such inherent heterogeneity associated with glycosylation pathways is integral to the biological function of glycans [1]. Notably, the loss of genes in glycosylation pathways often brings many devastating pathological consequences in humans, known as congenital disorders of glycosylation [2,3].

Glycans possess unique properties, distinguishing them from other biological molecules. First is the structural heterogeneity, which results from ten monosaccharides (glucose, galactose, *N*-acetylglucosamine, mannose, fucose, *N*-acetylgalactosamine, sialic acid, xylose, glucuronic acid, and iduronic acid) occurring in a human glycan composition, the anomeric configuration (α versus β), and a glycosidic linkage and branching between monosaccharides. Replacement of a hydroxyl group by another group (phosphate, sulfate, alkyl, acetyl, etc.) and their linkage to the parent aglycon (protein, lipid, etc.) increases glycan complexity. The heterogeneity is further amplified by assuming diverse proteins modified by glycosylation. Compared to proteins or nucleic acids, glycans exhibit extraordinarily higher numbers of structural isomers, e.g., a hexasaccharide has 10^12^ theoretical isomeric permutations [4]. Though most of these isomers may not exist, many glycans in the human glycome have already been described. In July 2024, the CSDB contained more than 33,450 distinct glycan structures, and new structures are continuing to be characterized. The second is the conformational flexibility. Glycans are flexible molecules, and the number of accessible conformations increases with glycosidic linkages. These existing conformers are in a dynamic equilibrium influenced by the environment [5]. Such flexibility allows glycans to adjust their 3D shape to physiological function requirements. For example, different conformers of a single glycan may interact with various ligands or receptors. Thus, the function defines the structure of glycans, not vice versa [6]. The third is the characteristic chemical structure. Glycan building blocks—monosaccharides, e.g., glucose, mannose, or galactose residues—have two faces: hydrophilic and hydrophobic. The top surface containing the hydroxyl group and the ring oxygen is more polar (hydrophilic), whereas the bottom surface, which consists of hydrogens, is less polar (hydrophobic). The bottom surface is involved in interactions with hydrophobic amino acids of the protein. Hydroxyl groups usually point to the solvent and interact with surrounding molecules. The chemical structure of glycans is often tuned for their function by replacing hydroxyl groups with, e.g., alkyls, phosphates, sulfates, and amines.

Consequently, glycans bear the biological information encoded by the monosaccharides’ sequence and glycosidic linkages and the glycan’s three-dimensional shape. The bioinformational content is decoded by lectins (proteins with carbohydrate-binding properties), antibodies, and pathogens. Glycan–lectin interactions are based on recognizing the glycan region called the carbohydrate determinant (often also oligosaccharide epitope, oligosaccharide antigens, and glycotope) [7] and are central for regulating their biological functions in various biological and pathological events. These interactions are also functionally relevant for multiple cellular processes [8,9].

The highly regulated repertoire of glycans covers the surface of the immune cells that also contain cell surface-located immune receptors and various carbohydrate-binding receptors (lectins). Almost all these compounds are glycoproteins or glycolipids [1,10,11,12,13]. Glycans display biological information that characterizes the cell’s type, are crucial for migrating immune cells, and contribute to sensing environmental signals with other molecules. Carbohydrate-binding receptors (CBRs) or glycan-binding receptors (GBRs) are involved in protein–glycan interactions responsible for pathogen recognition and modulate responses of innate and adaptive immune systems [14,15,16]. For example, immune cells use lectin–glycan binding to distinguish among self, modified self, and non-self glycomes. Thus, they recognize harmful pathogens and cancerous cells from healthy cells [17,18,19]. After recognizing danger, the immune system, through the cascade of steps, sends immune cells and molecules that promote inflammation [15,20,21,22]. The immune system response requires precise and balanced accuracy. If the necessary balance is violated, the response is either too weak and the body is infected or too strong, which may damage its tissues and organs.

Growing evidence demonstrates that glycans participate in many immune processes and that aberrant glycosylation severely impacts the pathology of various diseases, including autoimmune diseases, infections, inflammatory diseases, and cancer. As a result, glycan–GBP interactions represent a promising therapeutic target for treating multiple diseases. Therefore, it is no surprise that modulating interactions of glycan structures with their receptors in cells has potential for drug discovery. The focus in developing carbohydrate-based therapeutics includes selectins, galectins, Siglecs, glycosyltransferases, and vaccines and antibodies. Various strategies have been used. These include carbohydrate-based or carbohydrate mimetics, antibody-based therapeutics, GBP expression, and alteration of the biosynthesis of glycan determinants. Although many diverse agents that modulate glycan–GBP interactions have been developed, and despite considerable progress, only a few compounds have translated to clinics. Possibly the best-known inhibitors are the antiviral drugs zanamivir and oseltamivir [23] used for treating influenza. They function as transition state inhibitors of the influenza neuraminidase to block virus release from infected cells. Recent reviews on the progress in developing therapeutic agents targeting glycans and GBPs provide details that go far beyond the scope of this review [9,24,25,26,27,28,29,30,31].

In the last three decades, exciting progress has been made in understanding the fundamental roles of glycans in immune processes. A comprehensive understanding of glycan–GBR interactions is crucial for developing novel carbohydrate-based diagnostics and therapeutics in glycoimmunology. While several reviews [1,7,9,11,14,15,18,27,32,33] have described various roles of glycans in immune processes, it is justified to present a contemporary assessment of the advances in the field. This review will discuss the role of glycosylation in the modulation of immune cell interactions, pathogen recognition, and regulation of immune system response to inflammation and cancer from a “glycan’s eye view” perspective. The review will start with a brief description of glycosylation and glycosyltransferases involved in the biosynthesis of glycans associated with immunity processes, followed by a description of glycan-binding proteins decoding the bioinformational content of glycan, and a discussion on the role of glycans in immune cell trafficking, innate and adaptive immunity, antibodies, and carbohydrate-based vaccines.

## 2. Overview of Mammalian Glycosylation

### 2.1. N- and O-Glycosylation

Glycosylation of proteins in humans is the most abundant and structurally most diverse post-translational modification affecting proteins [34,35]. The biosynthesis of glycans involves various glycosyltransferases and glycosidases that, through various glycosylation pathways, produce different types of glycans, with *N*- and *O*-glycans being the most common [34].

**Figure 1:**
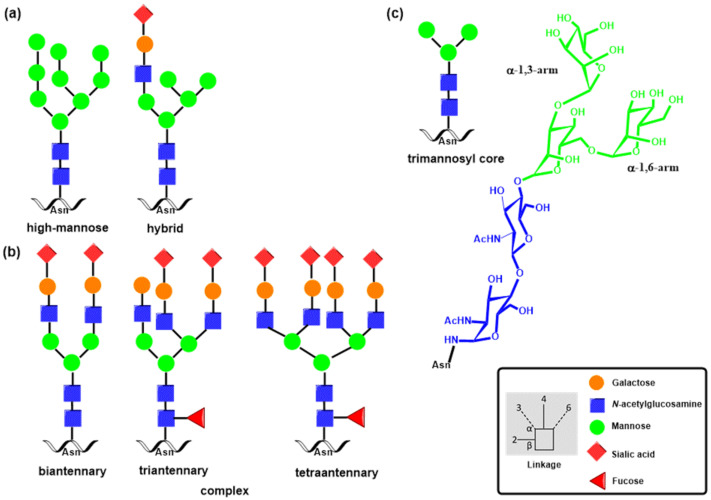
In the *N*-glycosylation process, *N*-glycans are linked by β-glycosidic linkages to the asparagine (Asn) side chain. A schematic representation of three major types of *N*-glycans biosynthesized in the ER and Golgi apparatus is shown: (**a**) high-mannose and hybrid types, (**b**) complex types, and (**c**) the core structure Man_3_GlcNAc_2_ pentasaccharide occurring in all *N*-glycan structures that can be differently terminated or extended with *N*-acetyllactosamine units, sialic acid residues, and other determinants. *N*-Glycan structures are shown using the symbol nomenclature for monosaccharides described in the box, together with the description of glycosidic linkages.

The formation of *N*-glycan in mammalian proteins commences by the transfer of the GlcNAc_2_Man_9_Glc_3_ glycan from a lipid precursor to the nitrogen atom of an Asn side chain in the ER linked via *N*-acetylglucosamine (GlcNAc) by oligosaccharyltransferase (OST) [36,37]. Then, glycosylation machinery employing various glycosidases and glycosyltransferases produces diverse structures encompassing complex and hybrid *N*-glycans (Figure 1) [38]. Finally, other glycosyltransferases produce glycan determinants that decorate *N*-glycan chains [7].

**Figure 2:**
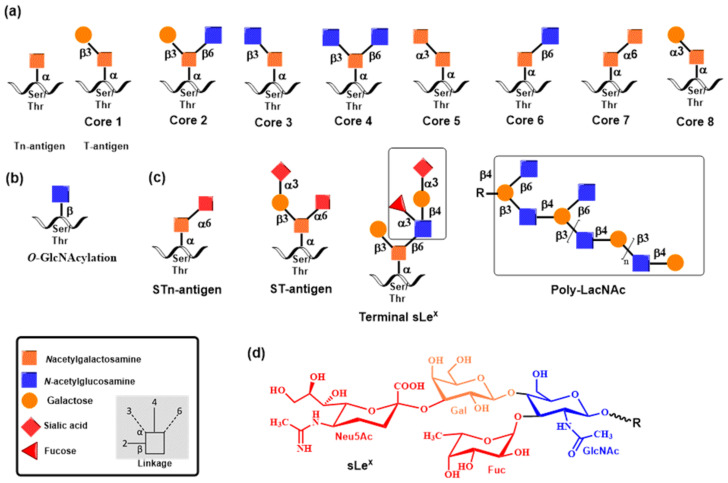
The schematic representation of *O*-glycans that are linked by α-glycosidic linkages of GalNAc to Ser or Thr side chains showing (**a**) core structures; (**b**) the structure formed by a single GlcNAc attachment to Ser or Thr in the O-GlcNAcylation process; (**c**) examples of the extension of the core; (**d**) the carbohydrate determinant sLe^x^. Structures in (**a**–**c**) are shown using the symbol nomenclature for monosaccharides.

Biosynthesis of mucin *O*-glycans commences by the transfer of *N*-acetylgalactosamine (GalNAc) to serine or threonine in the Golgi apparatus by the enzyme polypeptide *N*-acetylgalactosaminyltransferase family (ppGalNAcT) [39,40]. The action of ppGalNAcT enzymes generates the GalNAc-*O*-Ser/Thr (Tn antigen) structure shared by all *O*-glycan core structures. Then, core structures (Core 1–Core 8) are formed by specific glycosyltransferases that transfer galactose, *N*-acetylglucosamine, or *N*-acetylgalactosamine to the 3 or 6 positions on the *N*-acetylgalactosamine residue of Tn antigen by a β-linkage (Figure 2). These structures are further elongated or capped by fucosylation and sialylation by the action of fucosyltransferases and sialyltransferases, respectively. Monosaccharide residues of *O*-glycans can be further derivatized by replacing the hydroxyl with a sulfate or acetyl group. It is noteworthy that glycosidases are not involved in the biosynthesis of *O*-glycans. Both *N*- and *O*-glycans are often extended by poly-*N*-acetyllactosamine structures as a result of the consecutive action of two enzymes, acetylglucosaminyltransferase β-1,3-GnT and galactosyltransferase β-1,4-GalT. Recognition determinants frequently decorate the end of both glycan types for glycan-binding proteins, including Lewis and ABH blood group antigens [8].

Adding a single GlcNAc residue to Ser or Thr via a β-linkage is distinct from the above-discussed glycosylations. The biosynthesis is carried out by *O*-GlcNAc transferase (OGT) and is in delicate balance with the glycoside hydrolase, namely, *O*-GlcNAcase (OGA), that removes it. The transfer of the GlcNAc residue is not further extended. *O*-GlcNAcylation occurs in many proteins and is associated with various human diseases [41].

### 2.2. The Structure of Glycosyltransferases

**Figure 3:**
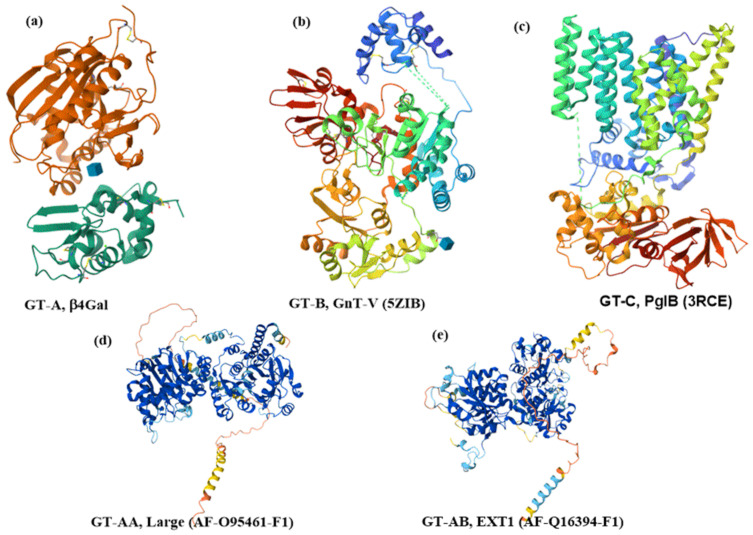
Glycosyltransferase folds. (**a**) GT-A fold structure, β4GalT, PDB entry 1NQI [44]; (**b**) GT-B fold structure, α-1,3-FucT, PDB entry 2ZNW [45]; (**c**) GT-C fold, PglB, PDB entry 3RCE [46]; (**d**) LARGE1 (AF-O95461-F1) [47]; (**e**) EXT1 (AF-Q16394-F1) [47].

Glycan biosynthesis involves a repertoire of glycosylation enzymes that act in a specific order along the ER-Golgi secretory pathway. Glycosyltransferases are the key players in the generation of glycan determinants (GTs, EC 2.4.x.y in the Enzyme Classification system), with many being membrane-bound glycoproteins. Glycosyltransferases create glycans by transferring one monosaccharide residue from activated nucleotide sugar donors to the hydroxyl group of specific acceptors. Based on their sequence similarities, glycosyltransferases are continuously clustered and have been classified into 137 families, including GT-1 to GT-137 [42,43], in the Carbohydrate Active enZyme database (CAZy, available at http://www.cazy.org/). Determination of their structure was relatively slow. However, the recent progress in the development of eukaryotic expression systems has led to an increased number of determined three-dimensional structures of GTs in the last two decades. The solved structures showed that despite the large number of families, their three-dimensional catalytic domain structures are surprisingly conserved and fall into three main classes: GT-A, GT-B, and GT-C (Figure 3).

GT-A fold (Figure 3a) is often regarded as a single Rossmann-like α/β/α domain fold, though distinct substrate-binding domains are observed [48]. In the GT-A fold, the central β-sheet is flanked by α-helices and a small β-sheet. The active site of enzymes belonging to the GT-A family usually contains the variable DxD motif, and glycosyltransferases require a divalent cation for their activity [49,50,51]. In ion-dependent GTs, the DxD motif and the negatively charged phosphate group of the nucleotide donor coordinate a divalent cation, usually Mn^2+^ or Mg^2+^. These electrostatic interactions significantly contribute to the binding of a donor. However, some GT-A enzymes lack the DxD motif, being a metal-ion-independent enzyme, such as Core 2 GnT glycosyltransferase [52,53]. Interestingly, the active site of the GT-A enzymes always contains an Asp or Glu residue, which serves as the catalytic base. Another structural characteristic of the GT-A superfamily is the presence of a disordered loop in the free enzyme, adopting the so-called “open” state. After binding the donor, the loop changes conformation to a “closed” state. This transition creates a lid over the donor substrate and a space for the accommodation of the acceptor [54,55,56].

The GT-B fold (Figure 3b) contains two Rossmann-like domains connected by a linker region facing each other. The catalytic site is located between these two domains, and GT-B enzymes are usually metal ion-independent enzymes. The binding of the nucleotide sugar donor in some of these GTs triggers a movement of two Rossmann-like domains, creating the productive active site. A nucleotide sugar binding site in the C-terminal region is relatively conserved in GT-B family members. In contrast, the N-terminal region, where various acceptors are accommodated, is more variable. It has been found that the donor moieties, ribose and diphosphate, often interact with glutamate and glycine-containing loops [57]. In GT-B enzymes, the diphosphate group of the donor is stabilized by a positively charged side chain of amino acids and a helix dipole [58]. Similarly to the GT-A enzymes, Asp or Glu residues are located in the enzyme active site to act as the base catalyst.

The first crystal structure of GT with GT-C fold (Figure 3c) has also been solved [59]. The GT-C fold consists of two domains, with the C-terminal globular domain involved in acceptor binding. The substrate’s binding site is located in the N-terminal transmembrane domain. It contains a variable DxD motif that probably creates a binding site for the diphosphate group of the lipid-linked oligosaccharide. The reaction center is at the interface between these two domains [60,61].

Recently, the structure of 215 human glycosyltransferases was investigated [47] using three-dimensional structures obtained from the AlphaFold Protein Structure Database [62]. Among the studied GTs, 133 comprised a single domain, with 82, 44, and seven belonging to GT-A, GT-B, and GT-C fold, respectively. Twelve GTs possess dual catalytic domains, with eight of them containing two GT-A domains; these have been called GT-AA (Figure 3d). The fold of four GTs having one GT-A fold and one GT-B fold has been called GT-AB (Figure 3e). The authors also discussed the topological arrangement of non-catalytic domains relative to the catalytic domain and their role in the properties of GTs.

### 2.3. The Catalytic Mechanism of Glycosyltransferases

**Figure 4:**
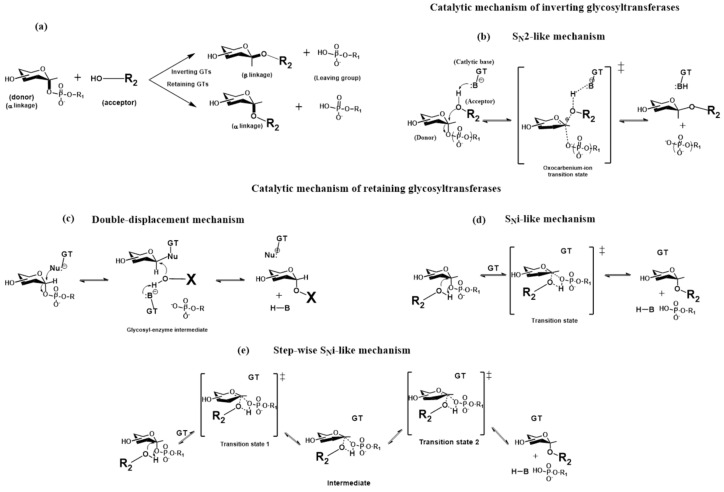
Schematic representation of (**a**) inverting and retaining reactions catalyzed by GTs; (**b**) S_N_2-like mechanism of inverting GTs; and catalytic mechanisms of retaining GTs, including (**c**) double-displacement, (**d**) S_N_i-like, and (**e**) step-wise S_N_i-like mechanisms. Transition states are marked with the double dagger (‡).

In the catalytic reaction, GT transfers a monosaccharide residue from a donor (nucleotide sugar) to the hydroxyl group of an acceptor via nucleophilic substitution. The stereochemistry of the anomeric linkage of the transferred sugar during this reaction can be either inverted or retained (Figure 4a), and glycosyltransferases are functionally classified as retaining or inverting enzymes. In the GT-A and GT-B families, GTs that proceed with either inverting or retaining mechanisms have been identified [35,58]. The efficiency of the glycosylation reaction depends on several circumstances, such as the availability of a particular donor nucleotide sugar in sufficient concentrations in a specific Golgi compartment, the required glycosyltransferase must be sufficiently expressed in a given Golgi compartment, and the acting glycosyltransferase can accommodate both the donor and acceptor in a productive conformation (Michaelis complex). The last requirement is crucial since the number of donors is restricted, and many GTs use the same donor for different acceptors. For example, UDP-GlcNAc is the donor for the biosynthesis of more than 20 different glycoconjugates [63]. In the last two decades, considerable effort has been made to understand the mechanical strategies glycosyltransferases use to form a new glycosidic bond, employing both experimental and molecular modeling tools. Significant progress has been recently reviewed [5,28,64,65]; therefore, only the main characteristics of various mechanisms used by GTs will be discussed.

The experimental data and molecular modeling studies support an S_N_2-like direct-displacement mechanism for inverting glycosyltransferases (Figure 4b). This leads to an inverted anomeric configuration via a single oxocarbenium ion-like transition state. The amino acid side chain in the active site, usually an aspartate, glutamate, or histidine, plays a crucial role in this mechanism by deprotonating hydrogen from the attacking acceptor hydroxyl group. These amino acids function as the catalytic base in the S_N_2-like direct-displacement mechanism.

Three different mechanisms were proposed for retaining glycosyltransferases. The initial, double-displacement reaction involving a covalently bound glycosyl-enzyme intermediate mechanism (Figure 4c) was based on the analogy with the mechanism used by retaining glycosidases. This mechanism involves two successive S_N_2-like reactions separated by a glycosyl-enzyme intermediate and leads to the retention of the configuration at the anomeric carbon. In this case, the first step is a nucleophilic attack (e.g., aspartic acid or glutamic acid side chain) on a donor’s anomeric carbon, leading to an intermediate inversion of anomeric configuration. In the second step, the nucleophile side chain (usually aspartate, glutamate) deprotonates hydrogen from the acceptor hydroxyl group, which attacks the anomeric carbon of the glycosyl-enzyme intermediate, leading to inversion and overall retention of stereochemistry. This mechanism is still under debate. Structural and biochemical studies on the retaining transferase LgtC [66] revealed the lack of a nucleophile within an appropriate distance from the donor. Subsequently, the authors proposed the S_N_i-like (or “internal return-like”) mechanism (Figure 4d). In this mechanism, the approaching acceptor nucleophile is partially deprotonated by the departing β-phosphate group of the donor nucleotide. The nucleophilic acceptor and leaving group are on the same side of the donor anomeric carbon, causing stereochemical retention at the anomeric carbon of the new glycosidic linkage. Later, another mechanism, the step-wise S_N_i-like (or “ion-pair”) mechanism (Figure 4e), was proposed [64]. The main difference is modifying the S_N_i-like mechanism by including an oxocarbenium ion intermediate, allowing a slight conformational change to facilitate the reaction.

### 2.4. Glycosyltransferases Associated with the Immune System

The functions of GBPs in the immune system influence the presence of required carbohydrate determinants. The carbohydrate determinants contain *N*- and *O*-glycans, usually located at terminal or elongated structures [7]. The biosynthesis of *N*- and *O*-glycans involves a set of glycosyltransferases that add monosaccharides consecutively, leading to the formation of core structures, followed by extension structures, and finally capping with terminal structures [34,38]. Glycosyltransferases engaged in the generation of ligands for GBPs include *N*-acetylglycosyltransferases, galactosyltransferases, *N*-acetylgalactosyltransferases, fucosyltransferases, sialyltransferases, and sulfotransferases that utilize UDP-GlcNAc, UDP-Gal, UDP-GalNAc, GDP-Fuc, CMP-NeuAc, and 3′-phosphoadenosine 5′-phosphosulfate (PAPS) as donors and will be briefly discussed in this section.

**Figure 5:**
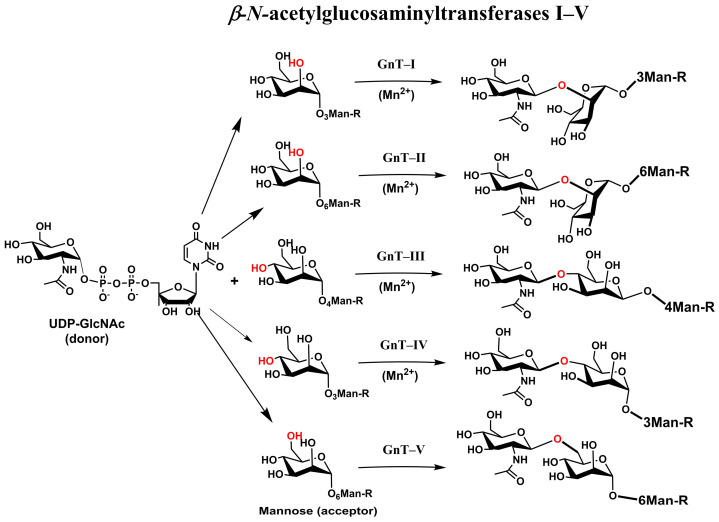
Schematic representations of the catalytic reaction of *N*-acetylglucosamyniltransferases I–V.

In *N*-glycans, five *N*-acetylglycosyltransferases [37,67,68,69] play a crucial role by adding a GlcNAc residue to the common core pentasaccharide Man_3_GlcNAc_2_Asn (Figure 5), generating *N*-glycan branches and resulting in bi-, tri-, and tetra-antennary structures (Figure 1). The biosynthesis of these structures commences with β-1,2-*N*-acetylglucosaminyltransferase I (GnT I), which forms a β-1,2 linkage of GlcNAc to the α-1,3-arm of the trimannosyl core of the Man_5_GlcNAc_2_ core. GnT-1 is an inverting, metal-ion-dependent glycosyltransferase of the GT-A family. The solved crystal structure [70] showed the presence of an EDD motif in the active site that binds the Mn^2+^ cation. Another critical step in the *N*-glycan branching pathway is the creation of the second β-1,2-GlcNAc linkage to the α-1,6-arm Man residue by β-1,2-*N*-acetylglucosaminyltransferase II (GnT-II), the Man3GlcNAc2 core. GnT-II is an inverting, metal-ion-dependent glycosyltransferase whose activity is abolished by the presence of a bisecting GlcNAc [71]. The 3D structure of GnT-II has been determined recently and revealed the presence of a DxD motif and GT-A fold [72]. The addition of GlcNAc to the β-mannose of the trimannosyl core through a β-1,4 linkage catalyzed by β-1,4-N-acetylglucosaminyltransferase III (GnT-III) [73] forms the so-called bisecting GlcNAc structure. It is assumed to be a key step in the biosynthesis of *N*-glycans. The bisecting GlcNAc residue is usually not elongated, and its presence inhibits the activity of GnTs and sialyltransferases [68].

The crystal structure of GnT-III has not yet been solved. However, based on the presence of the DxD motif, it was assumed that GnT-III adopts the GT-A fold [37]. Transfer of the GlcNAc residue to the α-1,3-arm of trimannosyl residue of the Man3GlcNAc2 core via the β-1,4-linkage is carried out by the β-1,4-N-acetylglucosaminyltransferase IV (GnT-IV) family [74]. The 3D structure of the GnT-IV has not yet been solved, and its functions are not fully understood and require further investigation. GnT-V catalyzes the transfer of a GlcNAc residue to the α-1,6-mannose of the trimannosyl core via the β-1,6-linkage to make the so-called “β-1,6-GlcNAc branch” structure [75,76]. Interestingly, GnT-V does not transfer GlcNAc to the α-1,3-mannose of the trimannosyl core. GnT-V is the inverting metal ion-independent enzyme. The crystal structures of GnT-V were recently determined [77,78] to show the GT-B fold, in contrast to previous GnTs that adopted the GT-A fold.

**Figure 6:**
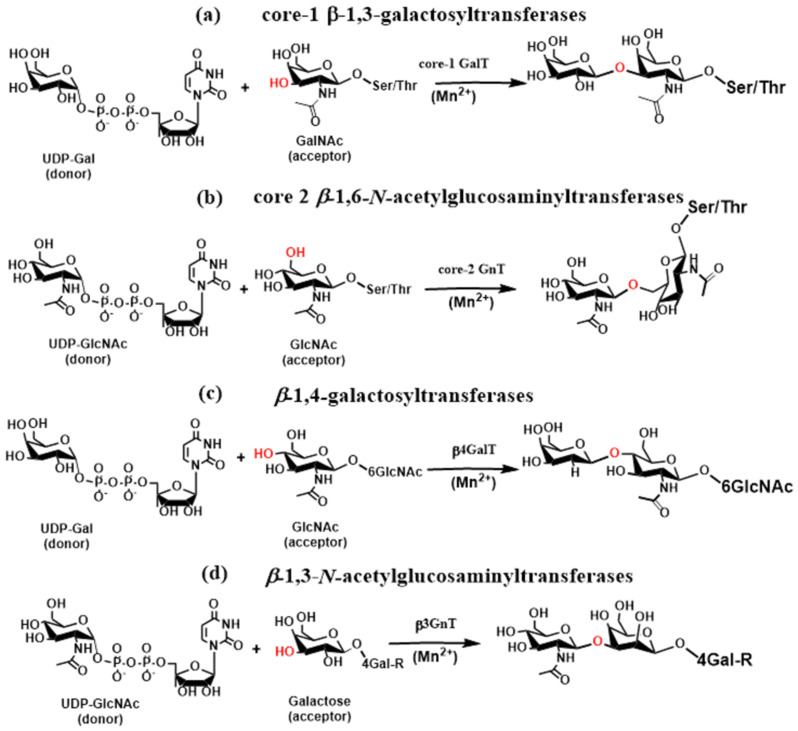
Schematic representations of the catalytic reaction of glycosyltransferases. (**a**) Core 1 β-galactosyltransferase, (**b**) core 2 *N*-acetylglucosamyniltransferases, (**c**) β-1,4-galactosyltransferases, (**d**) β-1,3-*N*-acetylglucosamyniltransferases.

In *O*-glycans, core 1 β-1,3-galactosyltransferases (core 1 GalTs, C1GalTs), core 2 β-1,6-*N*-acetyl glucosaminyltransferase (core 2 GnT, C2GnT), and core 3 β1,3-N-acetylglucosaminyltransferase (C3GnT) elongate Tn antigen (GalNAc-α-1-O-Ser/Thr) to create the core 1–4 structures that are further elongated or capped by the action of fucosyltransferases and sialyltransferases to generate glycan determinants associated with immune functions. Glycosyltransferase C1GalT [79] is the inverting metal ion-dependent enzyme that synthesizes core 1 disaccharides (T antigen) by adding galactose to GalNAc via a β-1,3-linkage (Figure 6a). Recently, the crystal structure of C1GalT1 was determined [80], revealing that C1GalT1 adopts a GT-A fold. The core 2 β-1,6-*N*-acetylglucosaminyltransferase [81] is a metal ion-independent inverting enzyme that creates key *O*-glycan branching and core 2 and core 4 structures [82]. Core2 GnT is a member of a group of β-1,6-*N*-acetylglucosaminyltransferases, and three isoforms (C2GnT1, C2GnT2, and C2GnT3) were identified [83]. One of three isoforms of core 2 GnTs transfers GlcNAc from UDP-GlcNAc to GalNAc of the core 1 structure via a β-1,6-linkage (Figure 6b). The crystal structures of leukocyte core 2 GnT were determined [52,53], showing the GT-A fold for the enzyme. However, a characteristic metal-binding DxD motif was not found. Instead, Lys and Arg were stabilized electrostatically, the leaving group nucleotide diphosphate [84].

The GlcNAc residue in bi-, tri-, and tetraantennary *N*-glycans and core 1 and core 2 *O*-glycans is often extended via the sequential action of β-1,4-galactosyltransferases (β4GalTs) and β-1,3-*N*-acetylglucosaminyltransferases (β3GnTs) to form an *N*-acetyllactosamine. Repeating their actions leads to a polylactosamine chain, a high-affinity galectin ligand. The β4GalT glycosyltransferase family contain seven members (β4GalT1-7) divided into four groups based on sequence similarity. The glycosyltransferase β4GalT is an inverting, metal ion-dependent enzyme with a GT-A fold that transfers a galactose residue from the donor UDP-Gal to acceptors GlcNAc, Glc, and Xyl, forming β-1,4-linkages (Figure 6c). In lactating mammary tissues, 4βGalT forms a heterodimer with a-lactalbumin and catalyzes the transfer of glucose from UDP-glucose to *N*-acetylglucosamine, forming lactose [85].

The Golgi inverting enzymes β-1,3-*N*-acetylglucosaminyltransferases [86] catalyze the transfer of a GlcNAc residue from UDP-GlcNAc to a galactose residue via a β-1,3-linkage, forming *N*-acetyllactosamine (Figure 6d). Up to now, seven members of this family (β3GnT2-β3GnT8) have been characterized [87]. Various crystal structures of β3GnT2 at different catalytic reaction stages have recently been determined [88]. The result shows that β3GnT2 adopts a GT-A fold and is metal ion dependent, having a DxD motif in the active site chelating divalent ion (Mg^2+^).

**Figure 7:**
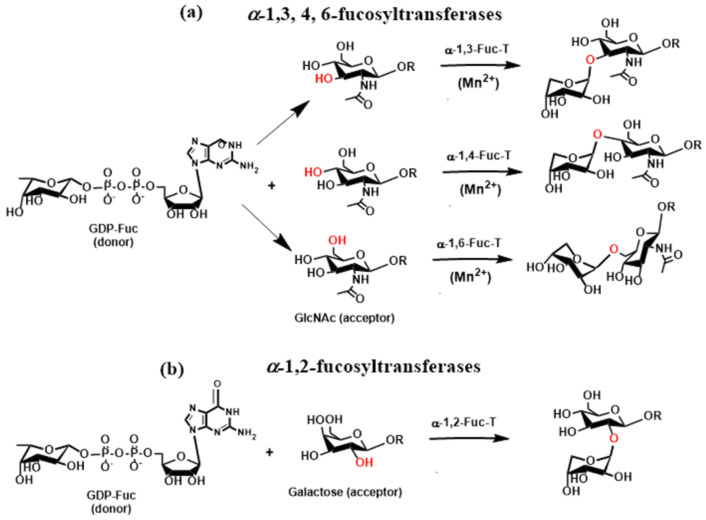
Schematic representations of the catalytic reaction of (**a**) α-1,3-, α-1,4-, and α-1,6-fucosyltransferases and (**b**) α-1,2-fucosyltransferases.

*O*- and *N*-linked glycans are also often terminated by Lewis and ABH blood group antigens [38]. They are attached to β-galactopyranose linked to GlcNAc or GalNAc via a β-1,3- or β-1,4-linkage catalyzed by β3-galactosyltransferase (β3GalT) or β4-GalTs as already mentioned above. The biosynthesis of these antigens requires the action of fucosyltransferases (FucTs) and sialyltransferases (STs). Fucosyltransferases (FucTs) are inverting enzymes that transfer the l-fucopyranose residue from the donor guanosine-5′-diphospho-β- l-fucopyranose (GDP-Fuc) to GlcNAc (Figure 7a) via α-1,3- and α-1,4-linkage in *O*-glycans (α-1,3-FucT and α-1,4-FucT) and α-1,6-linkage in *N*-glycans (α-1,6-FucT) as well as to galactopyranose (Figure 7b) via α-1,2-linkage in *O*-glycans (α-1,2-FucT) [89,90,91]. FucTs play a vital role in the synthesis of Lewis and HBO antigens. Transferring the α-linked l-fucose to GlcNAc of the terminal *N*-acetyllactosamine residue generates Lewis x (Le^x^) and Lewis a (Le^a^) antigens. In contrast, transfer to galactose leads to Lewis y (Le^y^) and Lewis b (Le^b^) antigens [92].

In *N*-glycans, the transfer of α-linked l-fucose to GlcNAc (Figure 7a) adjacent to Asn is carried out by the action of α-1,6-fucosyltransferase (FucT-VIII). The crystal structure of three FucTs was determined, namely *Helicobacter pylori* α-1,3-FucT [93], mammalian α-1,6-FucT [94], and bacterial α-1,6-FucT [95]. In all three FucTs, the 3D protein structure corresponds to the GT-B fold. The catalytic reaction of α-1,3-FucT requires the presence of metal Mn^2+^ or Mg^2+^ to proceed.

**Figure 8:**
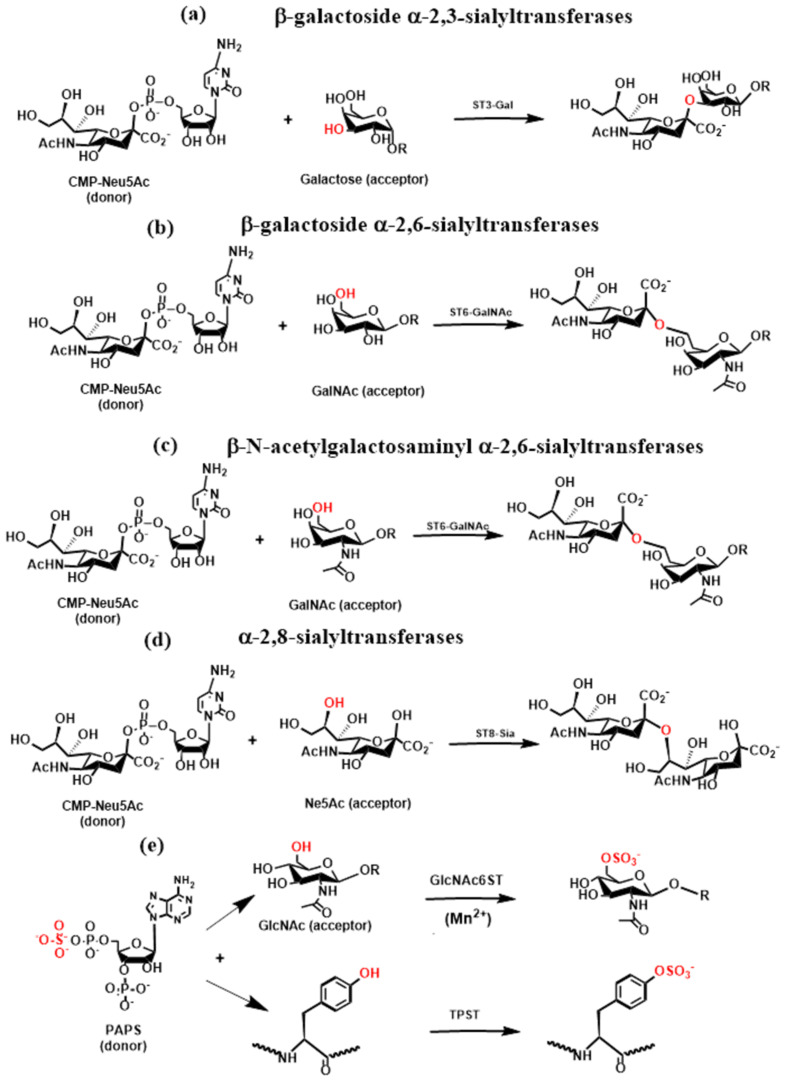
Schematic representations of the catalytic reaction of glycosyltransferases: (**a**) β-galactoside-α-2,3-sialyltransferases, (**b**) β-galactoside α-2,6-sialyltransferases, (**c**) β-*N*-acetylgalactosaminyl-α-2,6-sialyltransferases, (**d**) α-2,8-sialyltransferases (ST8Sia), and (**e**) sulfotransferases.

Many epitopes recognized by GBPs are sialylated. Their biosynthesis is finalized by the action of sialyltransferases that add *N*-acetylneuraminic acid (Neu5Ac, sialic acid) to the chain end of extended *N*- and *O*-glycans or their terminal structures [91,96,97]. Sialyltransferases are inverting, metal ion-independent enzymes that can adopt the GT-A or GT-B fold. They are grouped into β-galactoside α-2,3-sialyltransferases (ST3Gals), β-galactoside α-2,6-sialyltransferases (ST6Gals), β-*N*-acetylgalactosamine α-2,6-sialyltransferases (ST6GalNAcs), and α-2,8-sialyltransferases (ST8Sia). Sialyltransferases catalyze the transfer of sialic acid from a sugar nucleotide, namely, cytidine 5′-monophosphate sialic acid (CMP-sialic acid or CMP-Sia), to the terminal positions of *N*- and *O*-glycans (Figure 8). Sialyltransferases add sialic acids through an α-2,3-linkage to galactose (Figure 8a) or an α-2,6-linkage to galactose (Figure 8b) or *N*-acetylgalactosamine (Figure 8c) via an α-2,8-linkage to another sialic acid (Figure 8d), leading to polysialic acid structures [91]. Crystal structures of several STs were solved: ST3Gal [98,99,100], ST6Gal [101,102], ST6GalNAc [103,104], and ST8Sia [105]. The solved crystal structures of sialyltransferases revealed a diversity in the binding site and acceptor recognition.

*N*- and *O*-glycan sulfation is essential in tuning their biological activity and functions. Sulfation of carbohydrates is a post-translational modification carried out by Golgi enzymes, namely, sulfotransferases [106,107]. Sulfotransferases (STs) involved in the sulfation of glycoconjugates are membrane-associated enzymes that transfer the sulfuryl group (SO_3_) from a donor, PAPS, to an appropriate acceptor. Typical examples are the sulfation of the 6-hydroxyl of GlcNAc residue in L-selectin ligand sLe^x^, which is required for optimal binding and functioning in leukocyte rolling or the sulfation of tyrosine residues in PSGL-1 that significantly contributes to the high affinity of PSGL-1 with P-selectin [108]. The transfer of the sulfuryl group to the 6-hydroxyl of the GlcNAc residue (Figure 8e) is catalyzed by the *N*-acetylglucosamine-6-*O*-sulfotransferases (GlcNAc6STs). Although five isoenzymes of GlcNAc6ST were characterized [107], their crystal structure has not been determined yet. Sulfation of tyrosine residues (Figure 8e) is catalyzed by tyrosine protein sulfotransferase enzymes (TPST1 and TPST2) [109]. Recently, the crystal structure of human TPST2 was determined [110].

Many factors can influence individual glycosylation steps and final glycan structure, including the proper conformation of the acceptor and its location, the availability of donor substrates (sugar nucleotides), co-factors (e.g., Mn^2+^), location of enzymes, competing glycosylation reactions, and pH. Some pieces of evidence indicate that regulating the expression of different glycosylation enzymes is one of the mechanisms controlling glycosylation [11,34,111,112]. The expression of these enzymes depends on the cell’s developmental and physiological stage and tissue type [35].

## 3. Glycan Recognition in the Immune System

Mammalian glycans are highly regulated in the immune system, and their interactions with GBPs modulate various cellular mechanisms together with other molecules, thus playing fundamental roles in both innate and adaptive immune systems [11,14,113]. GBPs control immune responses in physiological and pathological situations by decoding differences between glycans on host, tumor, and pathogen cell surfaces. GBP–glycan binding is very sensitive and recognizes subtle variations in glycan structure. Glycan-binding proteins include C-type lectins (CTL) [114,115,116], galectins [117,118,119], and Siglecs [120,121,122]. They are expressed on immune cells or released into the cellular environment. GBPs recognize and bind carbohydrate determinants and thus function as pattern recognition receptors (PRRs) on various viruses, bacteria, fungi, and parasites [1,123,124,125].

### 3.1. C-Type Lectins

**Figure 9:**
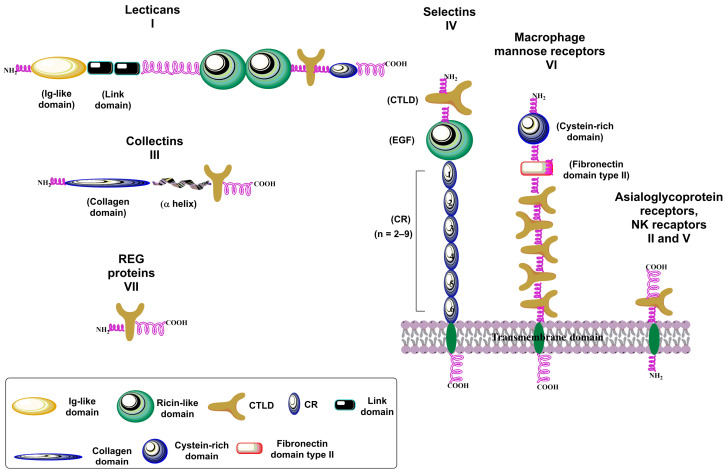
Schematic representation of vertebrate CTL domain architecture. Names and numbers of I–VII groups are indicated above the domain schemes.

The C-type lectins (CTLs) are a large superfamily of (glyco)proteins with diverse functions, initially defined for their ability to bind carbohydrates in a Ca^2+^-dependent manner [116] through a characteristic carbohydrate recognition domain (CRD). In C-type lectins, the CRD is more generally defined as the CTL domain (CTLD) because not all of the more than 1000 proteins belonging to CTLs have been observed to bind carbohydrates or Ca^2+^. Initially, based on their domain structure, vertebrate CTLs were divided into seven groups (I–VII) and later revised to include ten new groups (VIII–XVII) [115,126,127]. CTLs that bind carbohydrates are expressed as transmembrane glycoproteins or as secreted molecules in the extracellular space (Figure 9) and are fundamental in innate and adaptive immunity responses [14]. Among the seven traditional groups [115], group I, comprising lectinans, includes four large extracellular chondroitin sulfate proteoglycans (aggrecan, brevican, versican, and neurocan). For aggrecan, the highest affinity for fucose and galactose was observed among the sugars tested. The affinities for glucose and mannose were intermediate, and the affinity for *N*-acetylglucosamine was considerably lower [128]. Group II—asialoglycoproteins and DC receptors—encompass transmembrane proteins that bind to galactose-terminated oligosaccharides [129]. This group contains several heterogeneous subgroups: the asialoglycoprotein receptor (ASGR) subgroup (includes lectins ASGR and MGL) having a Ca^2+^ binding CTLD with primary specificity for galactose; the DC-SIGN subgroup (DC-SIGN, CD23, LSECtin) with a binding domain for mannose; the macrophage receptors subgroup (MCL, Mincle, DLEC, DCIR, DCAR, Dectin-2) with potential mannose-binding; the langerin and Kupffer cell receptor subgroup with binding affinity to mannose and galactose, respectively; and the scavenger receptor subgroup having a high affinity for galactose. Group III entails collectins that are soluble CTLs containing a collagen-like domain (MBP, PSP, CL-L1) and recognize ordered arrays of carbohydrates specific to pathogens. Group IV represents selectins, which are discussed in more detail below. Group V members, including NK-cell receptors (CD72, CD69, DCAL1, CLEC-1, CLEC-2, KLRL1, MDL-1, MDL-2, Dectin-1), are non Ca^2+^-binding II-type transmembrane CTLDs. Multi-CTL endocytic receptors (Group VI) are type I transmembrane proteins. Reg proteins (Group VII) are soluble proteins without a characteristic Ca^2+^ binding motif.

Although CTLs bind various ligands, monosaccharides are their primary targets [115], and most CLRs contain one or more CRDs. Multiple methods, including X-ray crystallography, biochemical, and molecular modeling, were used to investigate the mechanism of Ca^2+^-dependent binding of mannose and galactose [115,130,131,132,133]. The solved crystal structures revealed a similar arrangement of a monosaccharide in the binding sites. In the ternary complex formed among a monosaccharide, the Ca^2+^ cation, and amino acids within the CRD, critical interactions are noted for the monosaccharide’s hydroxyl groups at C3 and C4 [130,132]. Both hydroxyl groups are involved in three stabilizing interactions. First, they coordinate the Ca^2+^ cation; second, they function as the hydrogen bond acceptors from the NH_2_ side group of Asn residues; and third, they are hydrogen bond donors to the oxygen of acidic side chains. Additional stabilization is provided by hydrogen bonds between the carbonyl side chains from amino acids chelating the Ca^2+^ cation [115]. Consequently, the coordinated structure of the Ca^2+^ cation has eight oxygen atoms formed from two pentagonal bipyramidal structures.

**Figure 10:**
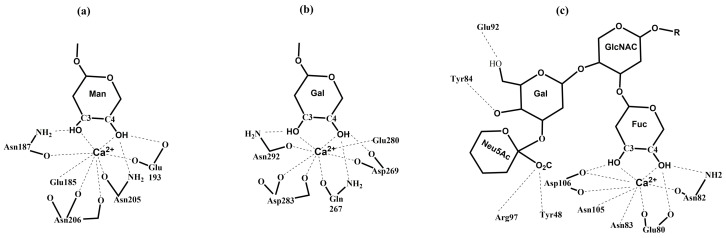
Schematic representation of Ca^2+^-dependent binding interactions of (**a**) the mannose-type ligand class with CLT based on crystal structures of MBP complexes [130,135]; (**b**) galactose-type ligand class with CLT based on crystal structures of MGL complexes [133,136]; and (**c**) binding interactions of sLe^x^ with E-selectin [137].

Carbohydrate specificity is determined by the specific amino acid residue motif within the CRD and the stereochemistry of the C3 and C4 hydroxyl groups. All carbohydrate-binding proteins of the CTL family are divided into the mannose-type ligand class or the galactose-type ligand class [116]. In mannose-type binding CTLs (they also bind GlcNAc, Fuc, and Glc), the sequence of conserved residues is the glutamic acid-proline-asparagine (EPN) motif. In galactose-type binding CTLs (also GalNAc), the specific motif is glutamine-proline-aspartic acid (QPD). Interestingly, the group of CTLs containing the EPN (or EPS) motif significantly prevails over those with the QPD motif. Though QPD or EPN moieties are primary structural characteristics for glycan binding specificity, other structural features of CRD are required for a stringent preference of mannose over galactose and vice versa [134]. Recently, the crystal structures of the CRD of the human macrophage galactose lectin (MGL) bound to GalNAc and the tumor-associated Tn antigen were solved [133]. The structures, together with saturation transfer difference NMR data, indicate a single binding mode for GalNAc in solution and show that CRD contains the characteristic glutamine-proline-aspartic acid (QPD) motif in an extended loop region and also the tryptophan-asparagine-aspartic acid (WND) motif in strand β4, which are responsible for Ca^2+^ coordination and substrate recognition. The interactions in mannose- and galactose-type LCTs are schematically shown in Figure 10. Of course, some additional interactions influencing their affinity to monosaccharides vary between CTLs, but they are not critical.

**Figure 11:**
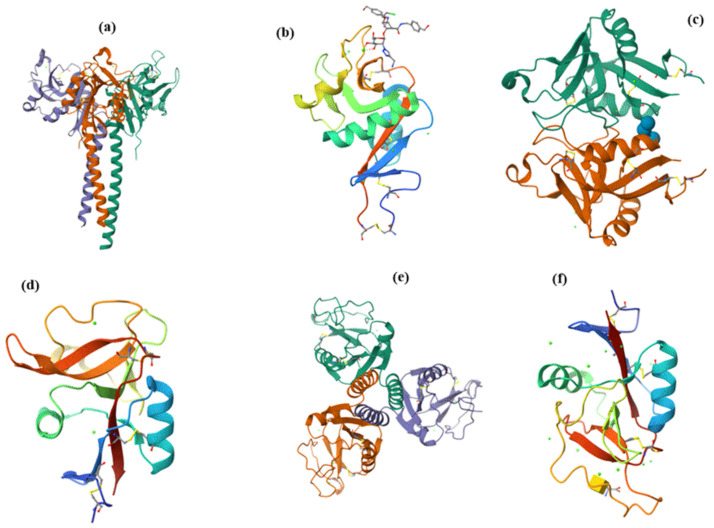
The 3D structures of selected CLRs: (**a**) homotrimeric langerin, PDB entry 3KQG [144]; (**b**) DC-SIGN, PDB entry 6GHV [145]; (**c**) dimeric dectin-1, PDB entry 2CL8 [146]; (**d**) CDR of CDIR, PDB entry 5B1W [147]; (**e**) homotrimeric MBL, PDB entry 1HUP [148]; (**f**) MGL, PDB entry 6W12 [133].

In addition to functioning as C-type lectin receptors (CLRs) that bind various pathogens recognizing particular glycan moieties, some C-type lectins may serve as adhesion, signaling, or antigen receptors. Since CLRs are present in antigen-presenting cells (APCs), they act as antigen-uptake receptors [138,139]. Most surface GBPs are glycoproteins and can function as PRRs by recognizing pathogen-associated molecular patterns (PAMPs) or cancer. In the following, some selected CLRs are described (Figure 11).

Langerin belongs to the type II group of CTLs expressed on Langerhans cells that make first contact with many microbes entering the body through the surface of the skin and mucosa [140,141]. The avidity and specificity of langerin are increased by forming a stable homotrimer (Figure 11a). The CRD has only one Ca^2+^ binding site and contains the EPN moiety. Consequently, langerin can recognize and bind mannose, fucose, and *N*-acetylglucosamine structures. However, only the homotrimeric langerin binds to glycoprotein ligands containing mammalian high-mannose oligosaccharides but does not bind to other complex glycan structures [142,143]. Langerin functions as a PRR and recognizes various pathogens through high-mannose structures.

DC-SIGN (dendritic cell-specific intercellular adhesion molecule-3-grabbing non-integrin) is a transmembrane glycoprotein belonging to the type II group of the CTL family. DC-SIGN is expressed on dendritic cells and macrophages, playing the crucial role of mannose-specific CTLs that recognize various pathogens [14,114,149,150]. Several recently solved structures revealed DC-SIGN structural features [131,145,151]. The structure of a DC-SIGN CRD [145] is shown in Figure 11b. DC-SIGN forms tetramers that are supposed to have the main impact on binding affinity by positioning the CRD in a position suitable for multivalent interactions with ligands. The present EPN moiety in CRD suggests that DC-SIGN, similarly to langerin, recognizes various pathogens [152,153,154].

Dectin-1 (dendritic cell-associated C-type lectin-1) is a natural killer (NK)-cell-receptor-like C-type lectin belonging to the type V group of the CTL family. It is present in myeloid cells, including macrophages, DCs, and neutrophils, and it is also present in specific lymphocytes [155,156,157]. However, in CRD, Dectin-1 lacks conserved residues required for Ca^2+^-dependent carbohydrate binding. Therefore, unlike other CLRs, Dectin-1 binds β-1,3- and β-1,6-linked glucans independently of Ca^2+^ with an unknown mechanism and is the primary receptor for β-glucans on leukocytes [158]. The solved crystal structures [146] revealed that Dectin-1 alone adopts a monomeric form but, in complex with glucans, forms a crystallographic dimer (Figure 11c) with a symmetrical binding site [146]. This finding suggests that oligomerization of Dectin-1 could increase avidity, leading to lattice formation. However, the authors could not assign any physiological importance to this dimer. It was also found that interactions of glucans with Dectin-1 induce the formation of molecular aggregates [159]. Dectin-1 participates in innate immune response as a pattern-recognition receptor recognizing β-1,3- and β-1,6-linked glucans found on the exterior of pathogens, including fungi, plants, some bacteria, and parasites [157]. The crucial role of Dectin-1 has been described in various diseases caused by multiple pathogens, though without describing putative ligands [160].

DCIR (dendritic cell immunoreceptor) is a type II membrane protein belonging to the type II group of the CTL family [161]. The crystal structure of the CRD of human DCIR alone (Figure 11d) and in the complex with a biantennary *N*-glycan unit at 2.90 Å resolution have been determined [147]. Instead of an EPN (glutamic acid-proline-asparagine) motif, the carbohydrate-binding site of DCIR contains the unusual sequence EPS (glutamic acid-proline-serine), with the WND motif also contributing to the glycan binding. Human DCIR is present in various immune system cells, such as B cells, monocytes, and myeloid DCs [161]. The glycan specificity of DCIR has only recently been studied, and different results have been obtained. It was found that DCIR binds mannose and fucose and weakly interacts with glucose and *N*-acetylglucosamine but also weakly binds galactose and *N*-acetylgalactosamine [134]. It was observed that human DCIR also binds sulfated *N*-acetyllactosamine, sulfated lactose, biantennary *N*-glycan, mannotriose, trisaccharide Lewis a antigen (Le^a^), and tetrasaccharide Lewis b antigen (Le^b^) [162,163]. All these interactions were significantly affected by the DCIR glycosylation [163]. It has been shown that DCIR either promotes or mitigates immune responses to various infectious diseases [164,165], inflammatory-related pathologies [166,167], and autoimmune disorders [167,168,169].

MBL (mannose-binding lectin), or mannan-binding protein, is a soluble C-type lectin that belongs to the CTL family type III group–collectins. MBL is a significant constituent of the human immune system that links innate and adaptive immunity [170] and belongs to pattern recognition molecules (PRMs) of the lectin pathway [116,171]. MBL is an extracellular glycoprotein mainly produced by liver cells and forms a bouquet-like complex consisting of two to six homotrimers [116,172]. The homotrimer crystal structure [148] is shown in Figure 11e. The presence of the EPN amino acid sequence in CRD explains the Ca^2+^-dependent specificity of carbohydrate binding by MBL. MBL recognizes mannose, *N*-acetylmannosamine, fucose, and *N*-acetylglucosamine and their derivatives, but not galactose, galactosamine, *N*-acetylgalactosamine, and terminal sialic acids located on the surface of infectious agents [116,173,174]. As a result, MBL recognizes a wide range of contagious organisms.

MGL (macrophage galactose lectin), also called human macrophage lectin [175], is a type II transmembrane glycoprotein that belongs to the type II group of the CTL family, exclusively expressed by DCs and macrophages. A single CRD contains the characteristic glutamine-proline-aspartic acid (QPD) motif and the tryptophan-asparagine-aspartic acid (WND) motif in strand β4 that mediate Ca^2+^ coordination and substrate specificity [136,176]. As a consequence, MGL specifically recognizes monosaccharides Gal and *N*-acetylgalactosamine (GalNAc) and is the sole C-type receptor that recognizes terminal GalNAc residues, including the Tn antigen (GalNAc-α-1-*O*-Ser/Thr), the sialylated Tn antigen (Neu5Ac-(2→6)-α-GalNAc-α-1-*O*-Ser/Thr), and the LacdiNAc epitope (GalNAc-(1→4)-β-GlcNAc) [177]. Recently, saturation transfer difference (STD) NMR spectroscopy [178,179] revealed that the MGL CRD is flexible, and its conformation depends upon the structure of GalNAc-containing ligands. Recently, the crystal structure of the MGL carbohydrate recognition domain in complex with GalNAc and Tn antigen has been solved (Figure 11f), explaining the GalNAc preference over Gal at the molecular level [133,180]. MGL can recognize tumor-associated cells via the Tn antigen, which is abundant in cancer cells and thus stimulates antitumor immunity [181]. Since the GalNAc residue rarely occurs on pathogens, MGL recognizes only a few pathogens. There are indications that MGL modulates various types of immune responses, and depending on the antigen structure, MGL can activate or suppress the immune system [182].

### 3.2. Selectins

Selectins are cell membrane glycoproteins belonging to the type IV of the CTLs that bind glycans in a Ca^2+^-dependent manner [183]. Selectin-mediated adhesion plays a crucial role in the physiology and pathology of various biological events, including inflammation, infection, cancer, and immune cell surveillance [27]. In immune cell trafficking, selectins play a fundamental role in the multi-step leukocyte adhesion cascade, explaining leukocyte recruitment to immune organs or inflamed tissues [21].

**Figure 12:**
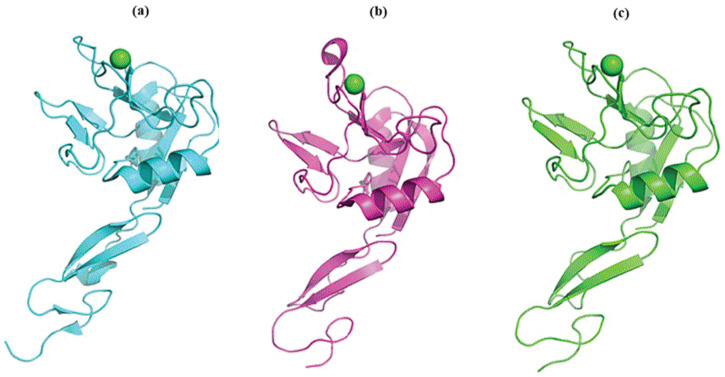
The X-ray crystal structures of CRD and EGF domains of the selectin family showing (**a**) P-selectin, PDB entry 1G1Q [187]; (**b**) L-selectin, PDB entry 3CFW [186]; and (**c**) E-selectin, PDB entry 1ESL [185]. The green sphere is the Ca^2+^ cation, an essential part of the carbohydrate recognition domain. The Ca^2+^ cation anchors the carbohydrate ligand to a selectin binding site; see details of sLex binding in Figure 10c.

The selectin family consists of three members: leukocyte (L)-selectin (CD62L), platelet (P)-selectin (CD62P), and endothelial (E)-selectin (CD62E). The P-, L-, and E-selectins exhibit similar primary sequences [184]. Crystal structures of three selectins with various ligands have been reported [137,185,186,187,188] and revealed a similar tertiary structure (Figure 12).

**Figure 13:**
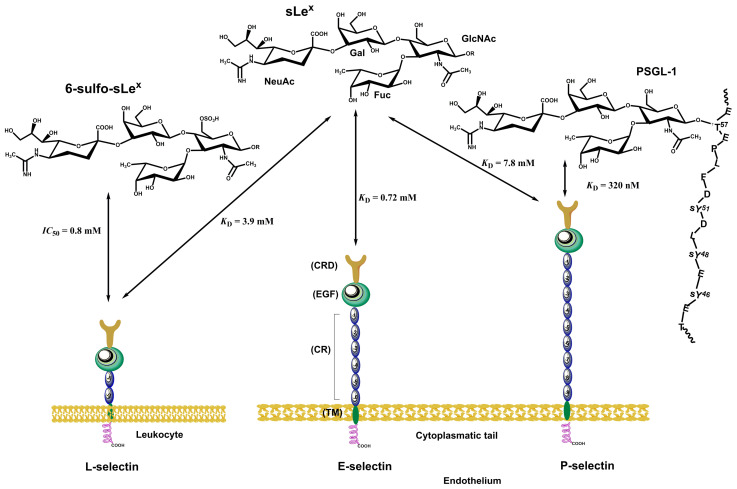
Schematic representation of P-, E-, and L-selectins and their carbohydrate ligands. All three selectins bind the minimal carbohydrate determinant sLe^x^ with low affinity [194]. Sulfation of GlcNAc enhances L-selectin affinity [195]. The binding affinity of P-selectin to PSGL-1 with all three sulfated tyrosines is considerably higher [108].

Selectins (Figure 9) consist of a short cytoplasmic tail, a transmembrane domain, a series of consensus repeat domains, an epidermal growth factor-like domain (EGF), and a carbohydrate recognition domain (CRD) [184]. They differ in the number of consensus repeats (CRs), with L-, E-, and P-selectin containing two, six, and nine CRs, respectively. Each consensus repeat has ~60 amino acids. P-Selectin is stored in α-granules of platelets and Weibel–Palade bodies of endothelial cells. It is released minutes after inflammatory activation, translocated to the cell surface, and shed and cleared from the cell surface after roughly 60 min [184,189]. E-Selectin is constitutively expressed by de novo transcription in the endothelia of the bone marrow vasculature within 3–4 h after inflammatory activation and is cleared approximately 24 h later [190]. L-Selectin is constitutively expressed on most circulating lymphocytes, monocytes, and granulocytes; located on finger-like projections called microvilli; and cleaved from the cell surface after cell activation [191,192]. All three selectins recognize and bind a common minimal glycan epitope α-Neup5Ac-(2→3)-β-d-Galp-(1→4)-[α-L-Fucp-(1→3)]-d-GlcpNAc-R (sialyl Lewis x, sLe^x^) and isomeric α-Neup5Ac-(2→3)-β-d-Galp-(1→3)-[α-L-Fucp-(1→4)]-d-GlcpNAc-R (sLe^a^) displayed at the end of various glycoconjugates [20,193]. The sLe^x^ binding is Ca^2+^-dependent, and crucial interactions for binding are schematically shown in Figure 4c. The affinity of three selectins toward sLe^x^ differs (Figure 13) and is in a millimolar range [*K*_D_(P-selectin) = 7.8 mM, *K*_D_(E-selectin) = 0.72 mM, and *K*_D_(L-selectin) = 3.9 mM] [194]. In the case of L-selectin, the sulfation of the GlcNAc residue of sLe^x^ at the 6 position increases the binding affinity [*IC*_50_(L-selectin) = 0.8 mM] [195].

**Figure 14:**
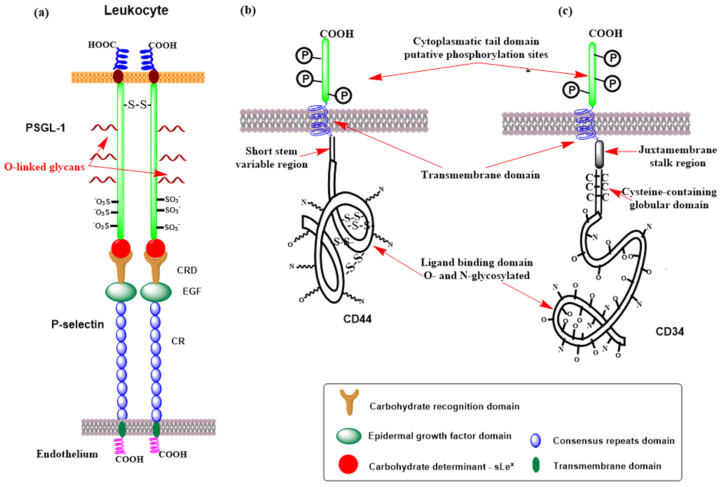
Schematic representation of some selectin ligands showing their domain architecture. (**a**) Homodimer of the pan-selectin ligand glycoprotein PSGL-1 in complex with two molecules of P-selectin. (**b**) E-Selectin ligand glycoprotein CD44. (**c**) L-Selectin ligand glycoprotein CD34. Reproduced with permission from reference [27].

Many glycoproteins and glycolipids are decorated by these determinants (sLe^x^, sLe^a^, or 6-sulfo-sLe^x^) and have been considered selectin ligands [196]. Notably, these glycoproteins and glycolipids often bind to selectins with a higher affinity, suggesting that the protein backbone and protein conformation play essential roles in selectin binding and selectivity [197]. Some of the ligands will be described next. The best-characterized pan-selectin ligand is P-selectin glycoprotein ligand-1 (PSGL-1, Figure 14a) [198]. PSGL-1 is a transmembrane sialomucin with the *O*-linked glycan determinant sLe^x^ and three sulfated tyrosine that forms a homodimer [198,199,200]. All three selectins recognize and bind PSGL-1 but with different affinities: *K*_D_ (P-selectin) = 320 nM [108] (Figure 13), *K*_D_ (E-selectin) = 15 μM, and *K*_D_ (L-selectin) = 5 μM [201,202,203]. PSGL-1 contains transmembrane, cytoplasmic, and extracellular domains [204].

The binding affinity of PSGL-1 is affected by sulfation of tyrosines (Tyr-46, Tyr-48, and Tyr-51). Model compound studies showed [202] that the sulfation of Tyr-48 (*K*_D_ ~ 6 μM) influences the binding affinity more than the other two sulfated tyrosines, which have the highest binding affinity (*K*_D_ = 0.65 μM). On the contrary, the non-sulfated model exhibited a considerably lower affinity (*K*_D_ ~ 25 μM). However, PSGL-1 is also the primary physiological ligand for L-selectin, with a lower affinity than P-selectin.

E-selectin binds glycoproteins containing sLe^x^, sLe^a^, and their modifications expressed in leukocytes [205]. However, it prefers fucosylated and sialylated glycoconjugates and binds sLe^x^ on L-selectin [206,207,208]. For E-selectin, several glycoproteins, including PSGL-1, ESL-1, CD43, and CD44, have been recognized as physiological ligands [209]. E-Selectin ligand-1 (ESL-1) is a transmembrane glycoprotein that recognizes E-selectin but not P-selectin. Many cells express ESL-1, including leukocytes; its localization is primarily in the Golgi apparatus [210,211]. An interesting difference in the biosynthesis of the carbohydrate determinant sLe^x^ between E- and P-selectin was found. While fucosyl transfer is carried out by fucosyltransferase IV (FucT-IV) in ESL-1, P-selectin utilizes fucosyltransferase VII (FucT-VII) [212]. Both ESL-1 and PSGL-1 are vital for the expression of E-selectin. In their absence, its expression is wholly abolished [213,214,215]. The sLe^x^ binding affinity *K*_D_ to E-selectin has been estimated to be between 107 μM and 1800 μM [194,216,217,218].

CD44 is a multifunctional transmembrane receptor (Figure 14b) that modulates cell adhesion and proliferation and is expressed in many mammalian cells [219,220]. Various CD44 isoforms (glycoforms) have a molecular weight between 80 and 220 kDa [221,222]. One of these isoforms, glycoform 90–100 kDa, was identified as a selectin ligand [223] that binds L- and E-selectin [224,225,226] and was named hematopoietic cell E- and L-selectin ligand (HCELL). HCELL is expressed by human hematopoietic stem and progenitor cells [227,228], where the epitope sLe^x^ is located exclusively at the end of *N*-linked glycans [229]. It is also expressed in some malignant cells [228,230,231] and classical human monocytes [232].

L-Selectin is a transmembrane glycoprotein expressed on most circulating leukocytes and characterized as a tethering/rolling receptor [191,233] The essential feature for L-selectin recognition is the GlcNAc sulfated sLe^x^ epitope (6-sulfo-sLe^x^) that is presented on numerous glycoproteins [183,233,234]. An exclusive characteristic of L-selectin is the presence of the sLe^x^ determinant, which is the ligand for E-selectin [235]. The primary function of L-selectin is as an adhesion receptor for ligands on the vascular endothelium. Recently, several basement membrane proteoglycans, including members of the heparan, dermatan, and chondroitin sulfate proteoglycan families, were also detected in extravascular locations as putative L-selectin ligands [236,237,238,239], but their function is not fully understood. Various L-selectin ligands have been recognized on the high endothelial venules (HEVs) of peripheral lymph nodes, including addressin (PNAd), CD34, glycosylation-dependent cell adhesion molecule (GlyCAM-1), mucosal vascular addressin cell adhesion molecule-1 (MAdCAM-1), podocalyxin-like protein, and spg200 [240,241,242,243]. These ligands are sialomucins, where proper carbohydrate determinants are *O*-linked to protein backbones by post-translational modifications. CD34 is a transmembrane phosphoglycoprotein (Figure 14c) expressed on endothelial cells. However, only the form located in the HEV is properly glycosylated for binding L-selectin [234,244,245]. L-Selectin affinity for CD34 is assumed to be similar to that of PSGL-1 [246]. Another potential L-selectin ligand is MAdCAM-1 (mucosal addressin cell adhesion molecule-1), an endothelial cell transmembrane glycoprotein containing a mucin-like region that displays an L-selectin glycan determinant [247]. MAdCAM-1 supports lymphocyte tethering and rolling through interaction with L-selectin and the α4β7 integrin. In patients with active or chronic intestinal inflammatory diseases, overexpression of MAdCAM-1 was observed, suggesting a role for MAdCAM-1 in lymphocyte rolling to the gut [248].

### 3.3. Siglecs

**Figure 15:**
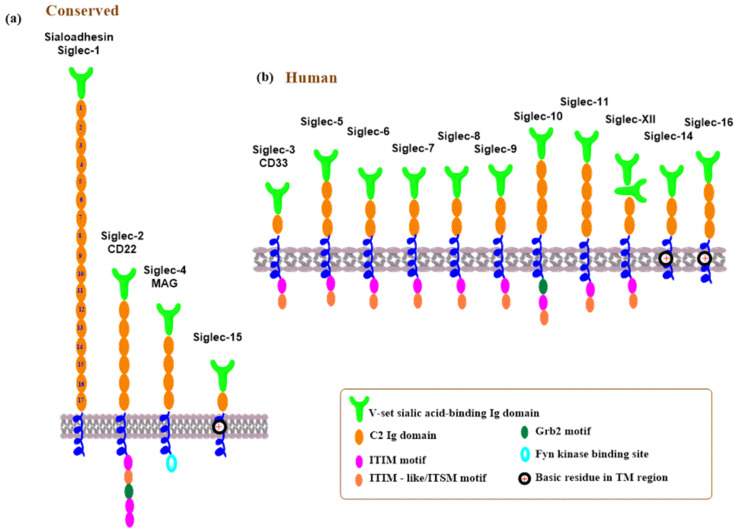
Siglec family proteins in humans. Schematic representations of domain structures of (**a**) four Siglecs conserved in all mammals and (**b**) eleven CD-33-related Siglecs.

Siglecs are transmembrane glycoproteins belonging to the immunoglobulin (Ig) superfamily of lectins [249,250]. They are primarily found in the cells of the immune and hematopoietic systems [120,121,122]. A schematic representation of the human Siglec family is shown in Figure 15. Structurally, Siglecs contain an extracellular region, an N-terminal V-set Ig domain that recognizes sialic acid, and a varying number of C2-set Ig domains. This is followed by a transmembrane domain and a carboxy-terminal short cytoplasmic tail frequently having tyrosine-based activating or inhibiting signaling motifs.

The human Siglecs family contains 15 members (Figure 15) and is divided into two groups: the evolutionarily conserved Siglecs and rapidly evolving CD33-related Siglecs [120,249,250,251]. The conserved Siglecs share about 25–30% structural similarity between vertebrates and include sialoadhesin (Siglec-1), CD22 (Siglec-2), MAG (Siglec-4), and Siglec-15 (Figure 15a). In humans, the members of the CD33-related Siglecs are numbered as CD33 (Siglec-3), Siglec-5-14, Siglec-16, and Siglec-17 (Figure 15b) [252]. An exception is Siglec-XII, which contains two N-terminal Ig-like V-set domains lacking the arginine residue required for sialic acid binding [253]. Siglec ligands encompass glycoconjugates decorated by sialic acids, and interactions between Siglecs and sialylated glycans modulate immune system response in health and diseases, engaging various signaling pathways. They are implicated, among others, in inflammatory diseases [121,254,255] and modulation of cancer cell invasion [251,256].

**Figure 16:**
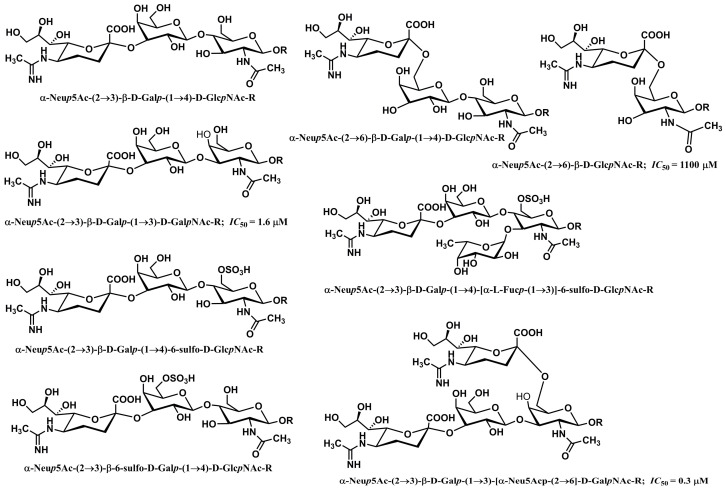
Schematic representation of selected natural Siglec ligands. The listed *IC*_50_ values for Siglec-4 (MAG) are from reference [260].

Siglecs containing the immunoreceptor tyrosine-based inhibition motif (ITIM) or immunoreceptor tyrosine-based switch motif (ITSM) participate in cell signaling and can trigger both inhibitory and activating signals [250,257]. A typical representative is Siglec-2 (CD22) [258]. A range of sialylated glycans is recognized as ligands by Siglecs [25,120,259]. In human Siglec ligands, sialic acid (*N*-acetylneuraminic acid, Neu5Ac) is predominantly bound by the α-2,3 and α-2,6 linkages to galactose in various oligosaccharides, and selected examples are shown in Figure 16. Siglecs show a low affinity for sialic acid (*K*_D_ in an mM range), and functional interactions are achieved by clustering Siglecs and ligands or by heavily glycosylated glycoproteins [121,260,261]. Some Siglecs recognize their ligand with high specificity, which is influenced by the type and physiological stage of the cells on which they are expressed. Siglecs are expressed on the surface of immune cells, including innate immune cells, but their expression profile depends on the types of immune cells and physiological conditions [262]. Siglec binding to sialylated glycans triggers molecular and cellular responses that affect the immunological function of the cell [259,263,264,265]. A distinct preference of immune cells for binding sialoglycan ligands plays a crucial role in distinguishing between self and non-self. Interactions between Siglecs in immune cells and sialylated glycans modulate immune responses and can promote or mitigate host defense. These interactions can occur on the same cell (*cis* interactions) or with ligands on soluble sialylated glycoconjugates or other cells (*trans* interactions) [258,266]. Siglecs are essential in managing inflammation of the immune response to various pathogens. They prevent uncontrolled inflammation by binding to proper self-ligands, ligating inhibitory receptors. It is clear that Siglec recognition and binding to sialylated glycans on pathogens are, on the one hand, favorable for immune response; however, there are situations where these interactions serve to mitigate immune response. Many aspects of this duality remain unclear and need further investigation. A delicate, well-balanced action is required. On one side, sufficient interactions are necessary to allow inflammation that eliminates pathogens. Conversely, too strong interactions might have undesired effects by damaging tissue [250]. Sialylated pathogens could exploit the inhibitory function of Siglecs to evade immune responses via Siglec ligation [267,268,269].

### 3.4. Galectins

Galectins are a family of soluble proteins expressed by various cells that bind galactose-containing glycoconjugates [32,117,118,270,271,272]. Galectins decode information accumulated in glycoconjugate structures and use it in immune system processes. They are synthesized in the cytosol and then exported to the extracellular space, avoiding the ER-Golgi apparatus pathway or remaining in the cytosol. Therefore, unlike other GBPs, galectins are not glycosylated, do not contain a classical secretory signal motif, and are constitutively expressed in every cell type [270]. In the cytosol, galectins interact with glycans exposed from various damaged vesicles, with glycans found on the surface of invading microbial pathogens or with intracellular proteins via a glycan-independent mechanism [1,272,273]. In the extracellular space, galectins bind glycoconjugates on the cell surface or extracellular glycoconjugates [271,273,274].

**Figure 17:**
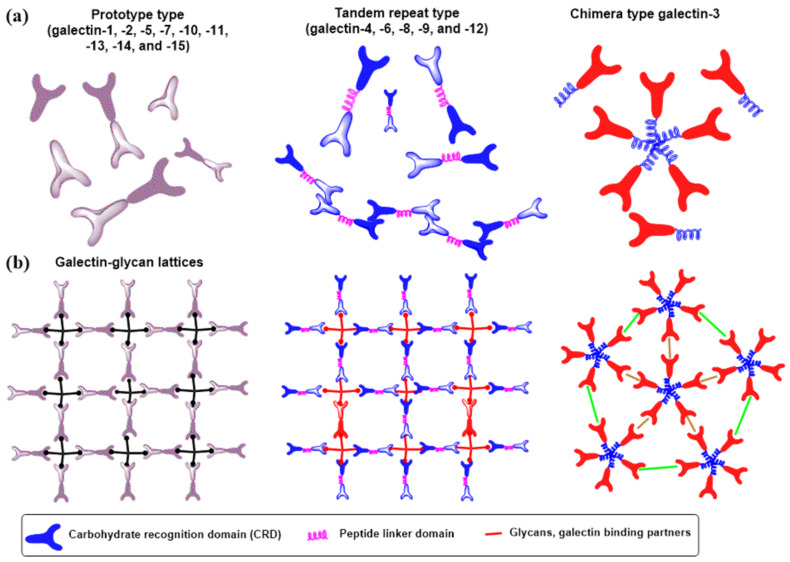
Schematic representation of (**a**) the three galectin types—prototype, tandem repeat, and chimera; (**b**) galectin–glycan lattices formed from the three galectin types.

Structurally, a family of human galectins contains more than 15 galectins that, based on their overall structure, are divided into three subfamilies (Figure 17a): prototype galectins, which include one CRD and exist in monomer–dimer equilibrium; tandem repeat-type galectins containing two CRD linked by a chain of up to 70 amino acids; and one chimera-type galectin-3, which has an unusual amino-terminal domain of proline- and glycine-rich sequences that can interact with intracellular proteins. In all galectins, CRDs consist of about 130 amino acids and adopt a common structural fold. Galectins typically exist as an equilibrium of various multimers (Figure 17a), from the homodimers of galectin-1 to the pentamer of galectin-2 or the large multimer of tandem-repeat galectins such as galectin-9 [270]. Carbohydrate determinants that decorate *N*- and *O*-glycans on glycoconjugates usually occur in clusters and can bind with galectin multimers, forming galectin–glycan lattices (Figure 17b). These lattices formed by cross-linking galectins with glycans on the cell surface or in the extracellular space generate homotypic or heterotypic aggregates that can also block the involved glycoconjugates from other bindings [275,276,277,278].

**Figure 18:**
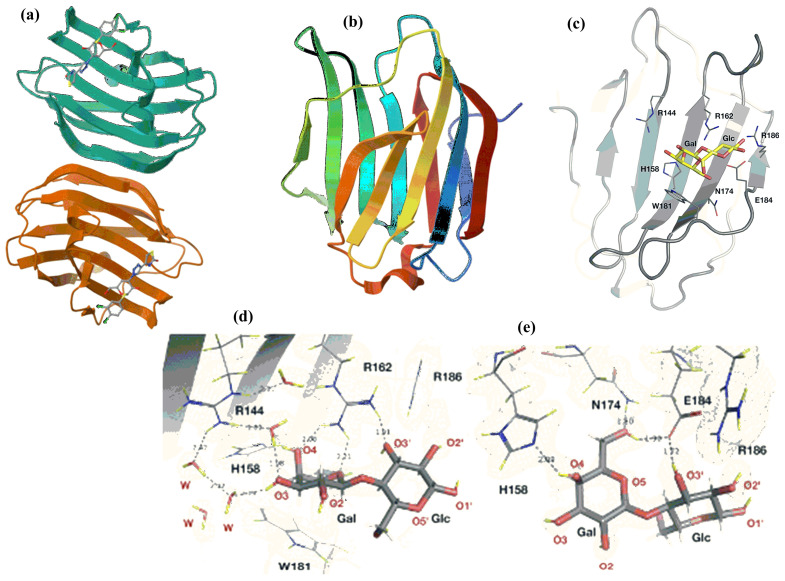
The X-ray crystal structures of the galectin family. (**a**) Galectin-1 in complex with a tiogalactoside derivative, PDB entry 8R74 [279]. (**b**) Apo human galectin-3 CRD at 1.08 Å resolution, PDB entry 3ZSL [280]. The crystal structure of galectin-3 in complex with lactose, PDB entry 6EYM [281] with (**c**) the lactose binding site in the C-terminal domain of galectin-3 overview. (**d**) Complex of galectin-3C with lactose. The nuclear density map is shown as a gray mesh. The lactose molecule is shown as thick sticks; protein side chains and water molecules that interact with them and lactose are shown as thin sticks. Deuterium atoms are colored yellow; hydrogen atoms on the lactose molecule are colored white. Relevant hydrogen bonds are shown as dotted lines with distances between the H atom and the H acceptor. (**d**) Interactions of GalOH4, GalOH6, and GlcOH3′ with His158, Gln174, Glu184, and Arg186 on the inner side of the binding pocket are highlighted. Arg162 is not shown for clarity. (**e**) Another view of the binding pocket rotated approximately 90° around the horizontal axis. Reproduced with permission from reference [281].

The crystal structures of various galectins or their domains in complexes with ligands were solved, and their binding characteristics were revealed. Figure 18 shows crystal structures of representative examples: galectin-1 in complex with a tiogalactoside derivative [279], apo-galectin-3 [280], and structural features of the CRD of galectin-3 in complex with lactose [281]. Galectins recognize the galactose residue. However, the binding affinity is weak [282,283]. Disaccharides containing galactose linked to Glc, GlcNAc, or GalNAc, such as lactose (β-d-Gal*p*-(1→4)-β-d-Glc*p*), *N*-acetyllactosamine (β-d-Gal*p*-(1→4)-β-d-Glc*p*NAc), lacto-*N*-biose (β-d-Gal*p*-(1→3)-β-d-Glc*p*NAc), and galacto-*N*-biose (β-d-Gal*p*-(1→3)-β-d-Gal*p*NAc), show higher affinity [284]. However, their *K*_D_ values are still in the low μM range, likely insufficient for biologically relevant interactions. For most galectins, the minimal ligand is *N*-acetyllactosamine. The crystal structures of the complex of galectin-3 with lactose revealed that the galactose residue plays a critical role in glycan binding. The structure also revealed non-covalent interactions between lactose and a series of conserved residues in CRD responsible for recognizing and binding ligands and explaining the galactose preferences over glucose by galectins [281,285,286].

**Figure 19:**
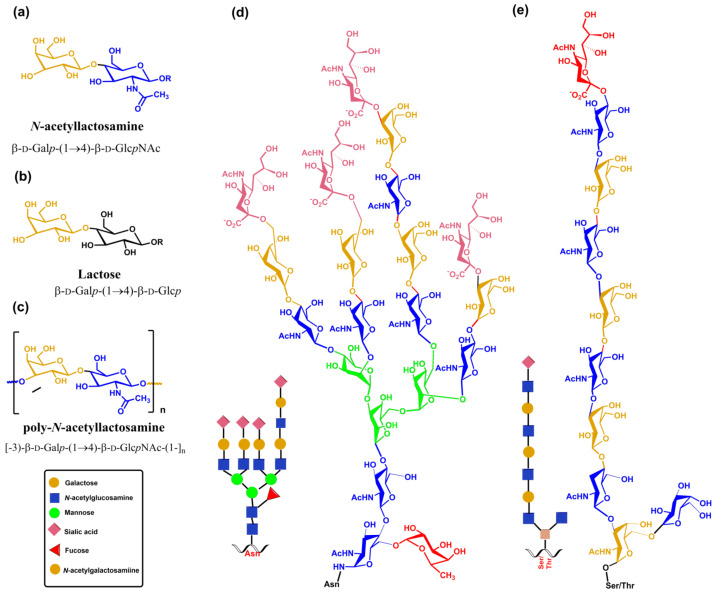
Schematic representation of galectin ligands: (**a**) *N*-acetyllactosamine, (**b**) lactose, (**c**) poly-*N*-acetyllactosamine, (**d**) tetranantenary *N*-glycan, and (**e**) polysialylated *O*-glycan. Tetranantenary *N*-glycan and polysialylated *O*-glycan are also shown using the symbol nomenclature for monosaccharides.

Soluble *N*-acetyllactosamine does not function as the physiological receptor of galectin; it usually binds to lactosamine sequences on complex *N*- and *O*-glycans, allowing multivalent binding. All galectins show higher binding for complex glycans (Figure 19) than disaccharides [287,288,289]. Structural features of *N*- and *O*-glycans, such as branching, repeating *N*-acetyllactosamine units, and sialylation in the 3 or 6 position of the terminal galactose, influence differences in the binding affinity. At least one galectin is expressed by every cell type, with the innate cells of the immune system loaded with galectins [32,270,290]. Since the *N*-acetyllactosamine moiety occurs in many *N*- and *O*-glycans, galectins can bind by non-covalent interactions with glycans on the same glycoprotein or the same or different receptors on the cell surface glycoproteins and extracellular glycoproteins and thus influence various cellular processes, including immune and inflammatory responses, autoimmune diseases, tumor development, and metastases [273,291,292,293,294].

Galentins were also discovered as pattern recognition receptors and signaling factors in innate and adaptive immunity [32,272,295]. Consequently, galectins can regulate host responses to various pathogens, recognizing and interacting with the non-self glycans of bacteria, viruses, fungi, and parasites [272,296]. Importantly, galectins, similar to selectins and Siglecs, can have opposite effects. Galectins can support or block interactions of pathogens with host cells. Some galectins have been observed to increase pathogen production, while others behave directly microbicidal for some pathogens [270,297,298]. Most of these studies were carried out using knockout mice and were discussed in a recent excellent review [272].

Many studies revealed that galectins exhibit two roles in modulating immune cell responses to pathogen infections [297]. However, despite an increasing number of experimental studies, the current understanding of the mechanism of action of galectins against pathogens is still fragmented [272]. A detailed discussion about the dual behavior of galectins can be found in recent reviews [272,292,299,300].

## 4. Glycosylation in Immune Cell Trafficking

The fundamental role of glycans in leukocyte recruitment emerged with the discovery of selectins, integrins, and their respective ligands in the last three decades. Leukocyte migration from the bloodstream into extravascular sites is the best-understood immunological function of glycosylation and is fundamental for innate and adaptive immune responses [11,20,21,183,301,302]. The circulation of leukocytes is a multi-step process and involves interactions between adhesion molecules and their glycan ligands, described as the leukocyte adhesion cascade. The cascade consists of several steps, from tethering of leukocytes from the bloodstream through selectin-mediated rolling, chemokine-triggered activation, and integrin-dependent firm adhesion to transendothelial migration into the tissue [22,302]. Selectin ligands play an essential role in the first two steps of the cascade, the tethering and rolling [303]. This process is mediated by reversible interactions of L-, P-, and E-selectins with PSGL-1 and their other ligands. P- and E-Selectin interactions with their glycan ligands initiate the tethering of free-flowing leukocytes on vascular endothelium. L-Selectin facilitates interactions between leukocytes via PSGL-1 in the later stage [304]. The repeating rolling interactions slow the leukocytes’ speed; consequently, the distance between leukocytes and the endothelial surface decreases [21,305]. E-Selectin interactions with the ESL-1 ligand are vital in slowing rolling speed, allowing a stable adhesion [213]. Rolling leukocytes along the endothelium proceed in blood flow under various shear stresses and require rapidly forming and dissociating the selectin–ligand complexes [303,306,307]. Selectin–ligand adhesion interactions are glycan dependent. The minimal recognition determinant recognized by the CRD of selectins is the terminal tetrasaccharide sLe^x^ and its isomer sLe^a^ [20,193]. Their biosynthesis is carried out by a sequential action of various GTs, including GnTs, GalTs, FucTs, SiaTs, and ST in the Golgi apparatus. It depends on the expression of GTs and the presence of their substrates. Any absence of GTs or substrates leads to imperfections in the ligand glycosylation structure and has detrimental effects on leukocyte extravasation to the site of inflammation or injury [183,301]. The aberrant glycosylation is also associated with different innate and adaptive immune responses [11] and various acute and chronic inflammatory diseases [232,308,309]. The intimate contacts between leukocytes and endothelium trigger chemical signals (chemokines, cytokines, and other inflammatory agents) that upregulate integrins, leading to firm adhesion and finally to endothelial transmigration to target tissues. In addition, it has been discovered that glycan structure plays a relevant role in the chemokine induction of leukocyte arrest [310] and integrin-mediated adhesion [311]. Lymphocytes also employ the cascade mechanism to migrate into peripheral lymph nodes and to the skin. Platelets use it to home to sites of hemorrhage [13,303], and tumor cells employ it during the metastatic cascade [312,313,314].

## 5. Glycosylation in Innate and Adaptive Immunity

In addition to the fundamental role of glycans in leukocyte trafficking, glycan–lectin interactions modulate all aspects of both innate and adaptive immune responses, including host–pathogen interactions, immunological recognition and activation, and self, non-self, and altered-self discrimination [1,11,12,14,15,16,17,18,19,33]. Innate and adaptive immunity, two immune system branches, use pattern recognition receptors [123] that detect invading pathogens and recognize and discriminate among self, altered self, and non-self-specific molecular structures. Almost all PRRs are glycosylated, including Toll-like receptors (TLRs) [315], NOD-like receptors (NLRs) [316], the major histocompatibility complex (MHC) [317], chemokine receptors [318], cytokine receptors [319], and T- (TCRs) and B-cell (BCRs) receptors [320]. Different glycans expressed on these immune cells fulfill various roles, though their roles are not yet fully characterized. Multiple studies have shown that glycosylation is fundamental for T-cell activation and differentiation [321] and revealed the critical role played by α-2,3-linked sialic acid [322] and complex *N*-glycans [323] in T-cell development. In addition, it has been demonstrated that *N*-glycans regulate T-cell activity and function [324]. *N*-Glycans lacking β-1,6-branching promote TCR clustering and signaling, which decreases T-cell activation thresholds and thus increases susceptibility to autoimmune diseases [325]. Since the GnT-V transferase catalyzes the formation of the β-1,6-branching, this finding suggests that GnT-V mediates T-cell functions. Experiments with GnT-I knock-out mice revealed that the lack of the β-1,2-linked GlcNAc significantly impaired B-cell development, supporting the role of complex *N*-glycans in the function of B cells [326]. Also, the α-1,6-fucosylation of the *N*-glycan core in the TCR and BCR by FucT8 affects their activity [327]. This supports the fundamental roles of glycans in T- and B-cell functions, early stages of T- and B-cell development, B cell-mediated production of antibodies, and pro- or anti-inflammatory properties of T cells [18,328].

Among PRRs, various GBPs represent a large family of compounds that identify environmental signals recognizing glycans on the surface of microorganisms such as bacteria, fungi, viruses, and parasites, known as pathogen-associated molecular patterns (PAMPs). Pathogens utilize different pathways to produce these glycans. The GBPs expressed in immune, antigen-presenting cells and innate immunity represent the first line of defense against pathogens [1]. GBP-lectin interactions were already discussed in Chapter 3. Here, only some specific examples will be mentioned.

### 5.1. Glycans in Bacterial Infections

Bacteria do not possess ER or Golgi compartments, and biosynthesis of *N*- and *O*-glycans by GTs can proceed in a lipid-mediated *en bloc* manner or OST-independent manner, with monosaccharide residues transferred to protein in a stepwise manner [329]. Although bacteria glycans are more complex than in eukaryotes, many organisms express similar sugars with standard functions [329,330]. On the other hand, bacteria contain some rare saccharides found only in bacteria, including, for example, 2,4-diacetamido-2,4,6-trideoxy-D-glucose (N, N′-diacetylbacillosamine or diNAcBac), 5,7-diacetamido-3,5,7,9-tetradeoxy-Lglycero-L-manno-nonulosonic acid (pseudaminic acid or Pse), 2,4-diacetamido-2,4,6-trideoxygalactose (DATDG), *N*-acetylfucosamine (FucNAc), and legionaminic acid [329,331]. Bacterial surfaces contain numerous glycoconjugates, including polysaccharide capsules and complex structures such as peptidoglycan and lipopolysaccharide (LPS), which are crucial for survival. Many of them function as PAMPs and are recognized by host PRRs, including CTL members langerin, DC-SIGN, Dectin-1, Dectin-2, and MBL [332]. An example is mannose-capped lipoarabinomannan (ManLAM) presented in pathogenic *Mycobacterium* spp. that can generate a pro-inflammatory or anti-inflammatory response [333].

Various human bacteria express sialylated glycans on their surfaces, mimicking the glycoconjugates in host cells; others obtain them from the host [265,334,335]. For example, the binding of Siglec-5 and Siglec-9 with sialylated *N*-acetyllactosamine displayed on Group B Streptococcus (GBS) induces inhibitory signaling and, together with attenuating the neutrophil oxidative burst, contributes to pathogen survival [336]. The role of galectins in viral infections is less transparent since information is rare. For example, galectin-3 stimulates the killing of *Helicobacter pylori* by macrophages [337]. On the other hand, galectin-3 facilitates bacterial replication and hinders the clearance of *Brucella abortus* [338].

### 5.2. Glycans in Fungal Infections

The surfaces of fungi are rich in *N*- and *O*-glycans, which mediate antifungal immune responses via interactions with CTLs. Fungi have evolved complex biosynthetic pathways in the ER and Golgi apparatus to synthesize a repertoire of glycan structures that differ from humans [339,340]. In yeast and other fungi, processing of the glycan chain in the Golgi involves only mannosyltransferases (ManTs) that add the mannose residue forming α-1,2-, α-1,3-, and α-1,6-linkages, leading to complicated hypermannosylated glycoforms. In the case of fungi, typical *O*-glycans are Ser or Thr-linked *O*-mannans [339,340]. The transfer of the mannose residue from dolichol-phosphate mannose (Dol-P-Man) to a particular protein is catalyzed by the GT-C enzymes *O*-mannosyltransferases (PMTs) [341,342]. These glycans are recognized by PRRs of immune cells, including langerin, DC-SIGN, mannose receptor (MR), Dectin-1, and Dectin-2. The dual role of galectins was also observed in fungal infections. For example, in infection by *Candida albicans*, it was observed that galectin-3 negatively affects neutrophil functions, and reduced galectin-3 expression increases human neutrophil reactive oxygen species (ROS) production [343]. Contrariwise, galectin-3 promotes an immune response against *Cryptococcus neoformans* infection in the spleen, brain, and lung tissues [344].

### 5.3. Glycans in Viral Infections

Viral envelopes are covered by various *N*- and *O*-glycans that fulfill various structural and functional roles: promoting expression, transport, and fusion; binding to cell surface receptors; and preventing antibody neutralization [345,346,347,348,349]. Viruses, contrary to other pathogens, utilize the host *N*- and *O*-linked glycosylation pathways to PTM of their proteins during replication. However, not all viruses follow the glycosylation pathways; some bud off early or traffic directly from the ER to the plasma membrane, leading to unmatured glycans [345]. For example, the hepatitis C virus (HCV) skips the ER, and mannose-type glycans dominate its *N*-glycan population [350]. However, some viruses, such as chloroviruses and mimiviruses, express enzymes that participate in glycosylation [351,352]. Viruses have evolved a strategy for molecular mimicry of host glycan determinants to evade immune detection and host colonization. On the other hand, some host glycans have diverse effects on the life cycle of viruses, including glycan shield-mediated immune evasion and enhancement of immune cell infection [349]. More than 200 human viruses have been recognized as causing human diseases of various severity [353]. Many of them have been extensively studied, and the role of glycosylation in infection in four of them, namely influenza virus (IV), human immunodeficiency virus (HIV), severe acute respiratory syndrome-related coronavirus (SARS-CoV-2), and Ebola virus (EBOV), will be briefly discussed.

Regarding influenza virus, hemagglutinin (HA) and neuraminidase (NA) are the two main surface glycoproteins responsible for the survival and replication of the virus. Both glycoproteins have several *N*-glycosylation sites. Host receptors MGL, MR, and DC-SIGN are attachment sites for hemagglutinin for infectious virus entry by recognizing *N*-glycans expressed on the HA surface. It was found that glycosylation of HA is vital for the pathogenicity of the virus [354] and influences NA functions. It was observed that NA regulates the activity of hemagglutinin by removing neuraminic acids that shade the receptor binding sites from its neighborhood [355]. Recently, the crucial roles of *O*-glycans have been revealed; however, they are not fully understood [347]. For example, it has been shown that biosynthesis of *O*-glycans is increased after IV infection by enhanced expression of a polypeptide GalNAc-transferase (ppGalNAcT, GALNT), leading to better virus replication [356]. The importance of glycans in IV infection is emphasized by the development of antiviral drugs (oseltamivir, zanamivir, and peramivir) that block the release of viruses from infected cells by blocking the enzymatic function of NA [357].

Human immunodeficiency virus (HIV) attacks immune cells, leading to weakened immunity to various bacterial infections and cancer development. Envelope glycoprotein (Env) subunits GP120 and GP41 are highly glycosylated and play a significant role in HIV attachment to host cells, fusion, virus entry, and infection of cells [347]. On average, GP120 contains 25 potential *N*-glycosylation sites with an interval of between 13 and 33 [358], occupied mainly by high-mannose *N*-glycans. It was found that removing glycans in the V1/V2 domain diminished the infectivity of the virus [359]. For human HIV infection, interactions between virus *N*-glycans with the host GBP, including langerin, DC-SIGN, MBL, DCIR, and MR, play crucial roles in the maintenance and spreading of HIV infection [18]. It is considered that langerin, located on the skin and mucosa, creates a protective barrier against HIV-1 infection [360,361]. However, despite the similarity of langerin and CD-SIGN, there is a significant difference in their functions. While HIV-1 binding to langerin leads to the degradation of the virus, HIV-1 binding to DC-SIGN stimulates infection [361]. The role of the conserved *O*-glycosylation is unclear; however, it has been associated with protecting the virus against antibodies, thus contributing to infectivity.

Severe acute respiratory syndrome-related coronavirus (SARS-CoV-2) has caused severe acute respiratory diseases during the recent pandemic (COVID-19). The human SARS-CoV-2 virus encodes envelope, membrane, and spike glycoproteins heavily covered by glycans [362]. The spike glycoprotein is homotrimeric and associated with virus binding and cell entry into a host body. Moreover, the spike binds to its receptor ACE2 on the host cell before entry and infection of the cell. A specific spike feature is many *N*-linked glycans (between 22 and 38 potential *N*-glycan sites per monomer) containing complex mannose structures, some of which are fucosylated and sialylated [362]. Also, core 1 and core 2 *O*-glycan structures were identified in the SARS-CoV-2 variants and located close to the *N*-glycosylation sites [363], though their precise function remains uncertain. Some experimental data showed that *N*- and *O*-glycans only marginally contribute to virus–ACE2 binding but play an essential role in the virus entry. The attachment of the virus to ACE2 was considerably decreased by inhibition of the glycan biosynthesis [364]. The virus glycans are recognized by CTLs of the host immune cells, including DC-SIGN and MGL, with conflicting results. In one study, the binding of DC-SIGN with virus glycans was associated with reduced infection by the blockage of viral entry [365], and higher levels of DC-SIGN on monocytes correlated with better prognosis [18]. In another study, it was suggested that DC-SIGN facilitates virus entry into human tissue. An increased level of DC-SIGN was observed in blood dendritic cells in severe SARS-CoV-2 patients, and it was associated with a higher expression of pro-inflammatory cytokines [366].

The Ebola virus is a highly pathogenic virus in humans, which can cause serious hemorrhagic diseases with a high mortality rate [367]. Surface glycoprotein GP consists of trimers formed by heterodimers GP1/GP2 and is fundamental for host cell attachment, entry, and membrane fusion [368]. Recently, the GP structure and the structures of GP complexes with the anticancer drug toremifene and the painkiller ibuprofen have been determined [369]. The host furin cleaves GP into two disulfide-linked subunits, GP1 and GP2. The GP1 subunit is responsible for receptor binding and consists of a base, head, glycan cap, and mucin-like domains (MLDs) [368]. The GP2 subunit contains the fusion peptide and two *N*-glycan sites that influence GP expression, stability, and cell entry [370]. The GP1 subunit contains up to 15 *N*-glycosylation sites. They are located primarily in the glycan cup and MLD, shielding GP from binding neutralizing antibodies, and their interactions with DC-SIGN mediate the host cell attachment [371]. In MDL, numerous *O*-glycosylated sites were identified containing not only Tn-antigen but also core 1 and core 2 sialylated structures. In addition, two *C*-mannoslated glycan sites has been predicted. However, their biological functions remain to be clarified [372]. It has been observed that *O*-glycosylation and sialylation affect capsid formation [373]. All these findings show that *N*-, *O*-, and *C*-glycans alter GP antigenic surface.

Various viruses, including HIV-1, SARS-CoV-2, and Ebola virus (EBOV), utilize ligation Siglecs to escape immune defenses and promote viral dissemination. The critical role is played by sialoadhesin (Siglec-1). However, some other Siglecs contribute to viral infection [334,374,375]. In addition, it was observed that galectins-1, -3, and -9 are elevated in the serum of patients with SARS-CoV-2 infection [376,377,378], and it was suggested that glycans could facilitate virus entry into cells [379]. In the case of avian influenza A H5N1, no effect of galectin-3 was observed. However, knockout mice showed less inflammation in the lungs [380]. Regarding cytomegalovirus, galectin-3 has a protective effect on hepatitis and attenuated inflammation in the liver [381].

### 5.4. Glycans in Parasites

**Figure 20:**
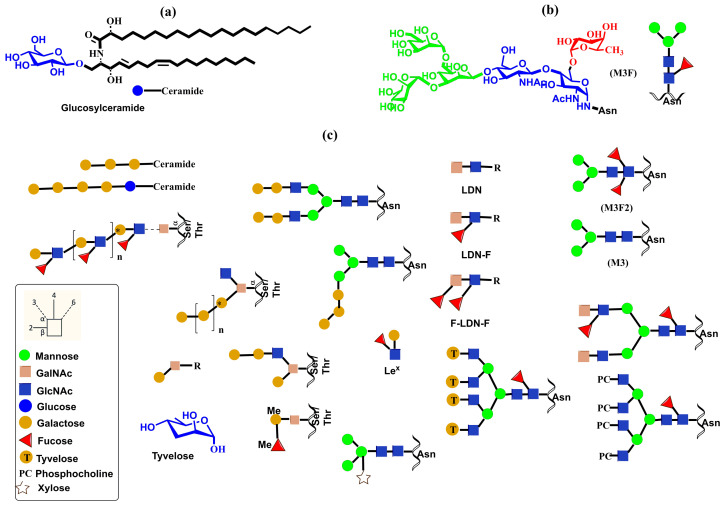
Schematic representations of glycan structures observed in parasites: (**a**) glucosylceramide, a typical representative of glycosphingolipids; (**b**) fucosylated paucimannosid glycan Man_3_GlcNAc_2_-Asn (M3F); (**c**) selected glycan structures observed in helminths and protozoa.

Parasitic infections caused by unicellular protozoans, multicellular helminths (worms), and ectoparasites are responsible for the lifelong suffering of millions of humans and belong to the leading causes of death [382]. Glycoconjugates cover the surface of parasites, creating a protective shield against the host defense and mediating human parasitic infections [382,383,384,385]. Various parasites also developed a glycan gimmickry strategy as an alternative to molecular mimicry [386], synthesizing host glycans interacting with the host GBPs to support their survival [387]. Though glycan biosynthesis in eukaryotes has standard features, various studies [383,384,385,388] revealed that many parasitic glycoproteins and glycolipids containing *N*- and *O*-glycans differ from host glycans (some are shown in Figure 20).

Glycans include unusual monosaccharides or monosaccharide modifications, such as tyvelose (3,6-dideoxy-d-arabinohexopyranose), 2-*O*-methylated fucose, and 4-*O*-methylated galactose or phosphorylcholine (PC). Another structural characteristic of parasitic glycans is the presence of β-d-GalpNAc-(1→4)-β-d-Glcp-Cer and β-d-Manp-(1→4)-β-d-Glcp-Cer instead of β-d-Galp-(1→4)-β-d-Glcp-Cer presented in mammals. Of course, parasites express a variety of the so-called ‘host-like’ moieties, including Le^x^, Le^b^, and β-d-GalpNAc-(1→4)-d-GlcpNAc (LacdiNAc, LDN) instead of the common *N*-acetyllactosamine (β-d-Galp-(1→4)-d-GlcpNAc, LacNAc, Figure 9), β-d-GalpNAc-(1→4)-[α-l-Fucp-(1→3)]-d-GlcpNAc (fucosylated LDN, LDNF), T- and Tn-antigens, and various glycosylphosphatidylinositols (GPIs). The unique feature of parasitic *N*-glycans is the prevailing abundance of the paucimannosidic (truncated) glycans, with the base composition Man_1–3_GlcNAc_2_-Asn (M1, M2, and M3) that can be terminated by a short mannosyl residue(s) or capped by various functional groups. These structures are often modified by adding the fucose residue via α-1,3- and α-1,6-linkages, forming mono- and bi-fucosylated paucimannosidic glycans (M3F, M3F2). All these parasitic glycans are highly antigenic. Interestingly, terminal *O*-GlcNAcylation modifies many parasitic glycoproteins. The most conserved general feature of parasitic glycans is likely the lack of sialylation that distinguishes them from glycans expressed by human cells.

Abundant glycans on the parasite surface are targets of the many hosts’ APCs, including macrophages and DCs, that recognize the glycans of parasites using PRRs such as CLRs and TLRs. Interactions between parasitic glycans and PRRs modulate innate and adaptive immune responses against parasitic infections. Among various CLRs expressed on immune system cells, DC-SIGN, MGL, and MR are associated with modulation of the immune response by interacting with glycans expressed on parasites [383,387,388]. The structure and properties of these CLRs are also discussed in Section 3.

DC-SIGN containing the EPN motif in CRD [131] belongs to the mannose-type ligand class of CTLs and, therefore, binds glycans containing terminal mannose or fucose residues such as Le^x^, Le^y^, LDNF, and high-mannose-type *N*-glycans [152,153,154]. DC-SIGN interactions with these antigens play a role in immune modulation in many parasites, including *S. mansoni*, *T. spiralis*, and *F. hepatica* [387]. The macrophage galactose-type C-type lectin (MGL) contains a QPD motif in CDR that is characteristic for recognizing glycans containing terminal galactose and GalNAc residues, such as the LDN, LacdiNAc, and the Tn antigens [177]. Human MGL potentially recognizes the glycan antigens of *S. mansoni*, *T. spiralis*, and *T. canis* [387].

The macrophage mannose receptor (MR) is a 175 kDa type I membrane glycoprotein first found in the liver. MR recognizes various mannose-containing glycans with the affinity determined by the terminal monosaccharide in order fucose > mannose ≥ *N*-GlcNAc > glucose, while the galactose affinity is substantially lower [389]. It is assumed that interactions of MR with mannose antigens on the surface of *T. spiralis*, *S. mansoni*, *A. suum*, and *Acanthocheilonema viteae* play a role in modulating immunity. However, this has not been demonstrated [387].

There are some CLRs present in the solution that interact with parasitic glycans. Typical examples are galectins, essential players in parasite infection, exhibiting two contrary roles. Galectins can facilitate parasite infection by functioning as a linker between host and parasite glycans or inhibit parasite infection by binding to host glycans and blocking the attachment of parasites [272,297]. *Leishmania* (L.) *amazonensis*, *Toxoplasma gondii*, and *Trypanosoma cruzi* infections represent parasites where galectins modulate immune responses [390,391,392,393]. Various studies indicate that interactions of parasitic antigens with DC-sign, MR, and MGL can proceed independently or in concerted action with other PPRs to induce immune responses [387].

An interesting mechanism of immune evasion is used by the parasite *Trypanosoma cruzi* (Chagas’ disease), which employs its enzyme trans-sialidase to trim sialic acid residues from the host and transfer them to the parasite coat mucin [394]. The resulting negatively charged coat of *Trypanosoma cruzi* has a protective effect. Several investigations of the role of galectins in parasite infections showed that galectin-3 decreases parasite load in *Leishmania amazonensis* [390] and *Toxoplasma gondii* infections [392]. On the contrary, the opposite effect was found in studies on the role of galectin-1 in *Trypanosoma cruzi* infection, where knockout mice showed lower mortality and parasite load in muscle tissue [391].

### 5.5. Glycans in Cancer

The changes in the glycosylation pattern of cancer cells have been used as a reliable hallmark of cancer for a long time [9,69,395]. Alterations in the glycosylation pattern of cells, particularly in glycan terminal motifs, correlate with cancer progression and the patient’s overall outcome [396,397,398,399]. Typical tumor-associated carbohydrate antigens (TACAs) observed in different cancers include sLe^x^, sLe^a^, β-(1,6)-branching in the *N*-glycans; the presence of the core-1 and core-2 structures with an increased presence of terminal sLe^x^, sLe^a^ tetrasaccharides in *O*-glycans; and truncation, sialylation, and fucosylation of both *N*- and *O*-linked glycans [395,400,401,402]. Patients with higher levels of TACAs usually exhibit larger tumors and metastasis and a lower rate of survival [395]. Many of these carbohydrate determinants, such as sLe^x^, Tn, and sTn, are present in almost all cancer types, and some others are typical for particular cancers. Some glycan antigens distinguish between cancer and healthy cells in the clinic, including CEA, PSA, CA19-9, and CA72-4, and recognize cancer antigens such as Tn, sTn or sLe^x^ [9]. Two molecular mechanisms have been suggested for the aberrant expression of cancer epitopes: ‘incomplete synthesis’ of pre-existing and ‘*neo*-synthesis’ of unusual carbohydrate determinants [403,404,405].

The migration of cancer cells from the primary tumor to metastatic states consists of several steps, known as the invasion-metastasis cascade, including leaving the primary tumor, circulation in the blood, extravasation and acclimation in a secondary site, and evading immune cells [406,407,408]. In all these processes, glycans play essential roles. Experimental data suggest that circulating tumor cells employ a mechanism similar to the inflammation cascade used by leukocytes [313,409,410,411], where interactions between E- and P-selectins and platelets and leukocytes mediate the tumor cells’ tethering and rolling on the endothelium. Selectins are also involved in interactions of cancer cells with endothelial cells, which are crucial for their extravasation and in transducing signals influencing the expression of several genes [411]. The involvement of selectins in cancer was investigated using various animal models. For example, the metastases of liver and breast cancers are reduced by down-regulating E-selectin [412]. Similarly, L-selectin knock-out mice showed a decrease in metastasis [413], and this effect was enhanced when both P- and L-selectins were deficient [414].

Besides involvement in the invasion-metastasis cascade, selectins recognize and kill cancer cells by the cancer-immunity cycle, supporting T cells to enter tumor sites [13,409]. Several other GBP families, including galectins and Siglecs, and CTLs, such as Dectin-1, DCIR, MGL, and DC-SIGN, are involved in cancer processes [415,416]. An overexpression of galectins is associated with cancer progression and contributes to cancer cell survival and metastasis [292,417,418]. For example, lowering the expression of galectin-1 suppresses vascularization in prostate cancer [419], while upregulated galectin-3 stimulates cell proliferation and invasion of tumor cells [420]. Quite the opposite behavior was observed for galectin-8 in colon cancer, where its expression decreased compared with healthy tissue, and galectin-8 acted as the suppressor of tumor cell migration [421]. Sialylated glycans belong to frequently occurring cancer antigens in all cancer types, and their interactions with Siglecs regulate immunosuppression and cancer evasion [422]. When sialylated glycans were trimmed of sialic acid by sialidases, tumor growth was diminished, supporting the above finding [423]. An overexpression of Siglecs was observed in various cancers, including Siglec-15 in colon, thyroid, kidney, and liver [424].

Tumor evasion of the immune system is facilitated by several identified inhibitory immunoreceptors, such as the cytotoxic T lymphocyte antigen-4 (CTLA-4) and programmed death cell protein 1 (PD-1) and its ligand PDL-1, which are named “immune checkpoint” molecules [415,425]. Immune checkpoint molecules interact with inhibitory receptors on lymphocytes, and blocking these interactions has been used in treating many types of cancer [426,427]. Various blocking antibodies entered clinical trials and clinics [31]. In particular, the inhibition of the PD-1/PDL-1 pathway by monoclonal antibodies (mAbs) has been a very successful therapy [426]. Several anti-PD-1 and anti-PDL-1 antibodies have been approved by the FDA, including pembrolizumab (Keytruda) from Merck & Co., nivolumab (Opdivo) from Medarex, cemiplimab (Libtayo) from Regeneron, atezolizumab (Tecentriq) from Genentech/Roche, durvalumab (Imfinzi) from Medimmune/AstraZeneca, and avelumab (Bavencio) from EMD Serono. Glycosylation of checkpoint molecules regulates structure, stability, and biological functions, especially the PD-1/PDL-1 interaction, which affects anti-cancer therapy. However, understanding glycosylation’s role is still limited [425]. Immune checkpoint inhibitors, monoclonal antibodies, adoptive cell transfer therapy, and tumor vaccines belong to immunotherapy treatments of cancer targeting glycans that have shown surprising results for some cancers [428,429].

The transmembrane *O*-linked glycoprotein mucin-1 (MUC1) has been extensively studied recently due to its overexpression in various cancer cells and use as a cancer antigen [430,431,432,433]. The MUC1 structure consists of an N-terminal subunit and a C-terminal subunit linked by stable hydrogen bonds [431]. The MUC1 is highly *O*-glycosylated in normal tissues with prevailing branched Core 2 structures and moderately *N*-glycosylated with glycans that shield the peptide core from proteolytic cleavage. MUC1 serves as a lubricant, moisturizer, and physical barrier to harmful environments for epithelial cells in healthy cells. In various epithelial adenocarcinomas, such as lung, liver, colon, breast, pancreatic, and ovarian cancer, MUC1 overexpression was associated with disease progression [434,435]. In cancer cells, the structures of the glycans undergo considerable change, leading to aberrant glycosylation [430,432], in which *O*-glycan structures are underglycosylated and highly sialylated. The Core 1 structures dominate due to the lack of the C2GnT activity that forms the Core 2 structure, adding GlcNAc by β-1,6-linkage to the Core 1 GalNAc [436]. Sialylation of *O*- and *N*-glycans is carried out by enzymes β-galactoside-α-2,3-sialyltransferases and β-galactoside α-2,6-sialyltransferases (Figure 19) that are overexpressed in various cancers [396]. These alterations in MUC1 glycosylation are linked to cancer invasion, metastasis, angiogenesis, apoptosis, and immune surveillance. MUC1 was observed to function as a pro-inflammatory factor in different cancers, including pancreatic, breast, and colorectal, utilizing the leukocyte adhesion pathway [437]. On the other hand, in multiple sclerosis, it was detected that MUC1 inhibits dendritic cells, thus having anti-inflammatory functions [438]. The structure and properties of MUC1 make it a good candidate for use in the clinic. It is used as a biomarker in gastric, colorectal, and pancreatic cancers, where its levels correlate with different stages and the prognosis of patients [439]. MUC1 also plays a relevant role in immunotherapy, which is used in antibody- and vaccine-based therapy [440].

## 6. Antibody Glycosylation and Vaccines

The enormous structural heterogeneity of glycans affects the antigenicity of glycans and provides a wide variety of glycan antigens that stimulate the production of antibodies in humans [30,31,441]. Glycan antibodies are used for various purposes in clinics and basic research. The structural specificity of complex glycoconjugates makes antiglycan antibodies a fundamental tool in detecting, purifying, and discovering glycan epitopes in various stages of a disease. Therapeutically, they can activate or suppress biological processes where glycans play crucial roles. However, compared to proteins, the number and diversity of suitable monoclonal antibodies to glycans are less available [441].

### 6.1. Antibody Glycosylation

**Figure 21:**
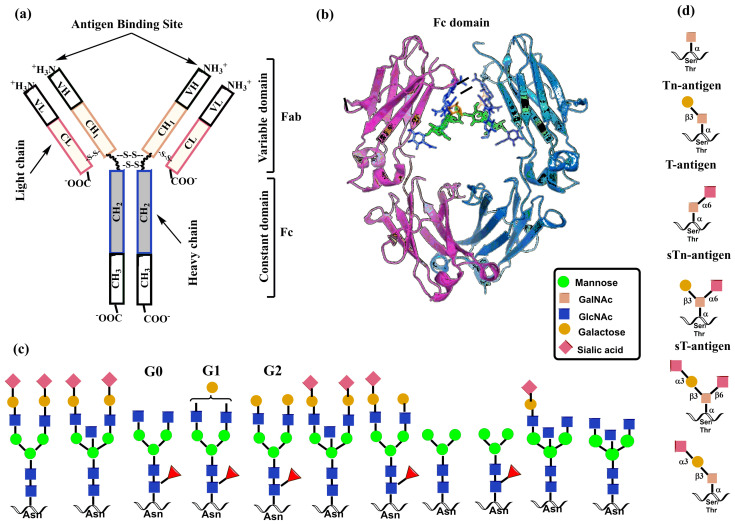
Schematic representation of (**a**) immunoglobulin monomer. (**b**) Ribbon diagram of the crystal structure of the Fc domain of IgG, PDB entry 3AVE [442]. Glycans are displayed in colored sticks. Selected (**c**) biantennary *N*-glycans and (**d**) *O*-glycans found on human Igs.

Antibodies (immunoglobulins, Igs) are Y-shaped glycoproteins (Figure 21a) that play a crucial role in promoting immune response to various pathogens [442,443]. In humans, five distinct classes have been identified: IgG, IgM, IgA, IgE, and IgD. IgG and IgA are divided into subclasses: IgG1-IgG4 and IgA1-IgA2. Immunoglobulins are heterodimer glycoproteins that consist of two heavy chains (H) divided into four domains (VH, CH1, CH2, and CH3) and two light chains (L) that consist of two domains (VL and CL). Antibodies consist of two functional domains. The constant Fc domain mediates interactions with various Fc receptors, and the variable Fab domain recognizes and binds antigens [444]. Recently, 3D structures of multiple Igs and their components have been determined at the atomic scale; see, e.g., references [442,445,446,447,448]. A flexible, nonstructural hinge region between the CH1 and CH2 domains links the Fc and Fab domains in IgG, IgA, and IgD. The two remaining antibodies, IgM and IgE, are connected by a more rigid domain. All antibodies are glycosylated and differ in the location and the number of *N*- and *O*-glycosylation sites. Glycans mediate their stability, secretion, immunogenicity, and function [443,444,449,450,451]. While IgG molecules have a single *N*-linked glycan site, IgM has five, IgD three, IgE seven, IgA1 two, and IgA2 five *N*-glycosylation sites. In addition, antibodies IgD, IgG3, and IgA1 bear multiple *O*-glycosylation sites [444,451].

Of note, *N*- and *O*-linked glycosylation affects antibody structure and functional behavior. These PTMs commence as in other proteins in the ER and continue in the Golgi apparatus. In immunoglobulins, biosynthesis leads to various *N*-glycan structures, and the prevailing is a classical biantennary core structure GlcNAc_2_Man_3_GlcNAc_2_ [450,451,452]. This structure may be further sequentially modified by adding fucose, galactose, a bisecting GlcNAc, and sialic acid, generating a repertoire of *N*-glycans [449,453,454,455]. Selected *N*-glycan structures found in the antibody Fc domains are shown in Figure 21c. All modifications of the core biantennary structure are responsible for *N*-glycan diversity and are associated with modulating immunoglobulin’s sensor affinity and enhancing the functionality of immunoglobulins [449]. The composition of *O*-glycans in the hinge region of immunoglobulins varies. The *O*-glycoforms usually consist of a mixture of truncated *O*-glycans with *N*-acetylgalactosamine linked to Ser or Thr, which is extended by a β-1,3-linked galactose and variable sialylation (Figure 21d) [456].

### 6.2. Antibody Glycosylation in Autoimmune Diseases and Infection

Altered glycosylation of immunoglobulins was documented in various immune-related processes, including autoimmune and inflammatory diseases [33,457,458]. Various changes were observed in IgG glycosylation with age and even sex [459,460]. Considerable changes in Ig glycosylation were also observed during infections [450,461], autoimmune diseases [458,462], and cancer [463,464]. For example, changes in galactosylation of IgG are associated with a variety of diseases, including systemic lupus erythematosus (SLE), inflammatory bowel disease (IBD), ANCA-associated vasculitis (AAV), immune thrombocytopenia purpura (ITP), fetal and neonatal alloimmune thrombocytopenia (FNAIT), and autoimmune liver disease [33]. A decrease in galactosylation was likely the first observation that linked rheumatoid and osteoarthritis arthritis (RA and OA) to glycosylation change in antibodies [465]. In healthy humans, three possible galactosylated *N*-glycans (Figure 21c) are in equilibrium: glycans having no galactose residue (G0)~with one galactose residue (G1) > two galactose residues (G2 [457]). Various studies have shown that the fraction of G0 glycans considerably increased in inflammatory diseases, and on the contrary, G1 and G2 glycan increases are linked with reduced inflammatory activity [466,467]. Similarly, the percentage of sialylated IgG glycans is lower during inflammation and autoimmune diseases than in healthy humans [457,468].

The role of *O*-glycans in antibodies is less understood than that of *N*-glycans. However, the autoimmune disease IgA nephropathy (IgAN) is associated with aberrant *O*-glycosylation [469,470]. In patients with IgAN, IgA1 antibodies possess a fraction of truncated *O*-glycans deficient in galactose residue [471,472,473]. Thus, instead of core 1 structures (T-antigen), prevailing *O*-glycans in IgA1 are the Tn-antigen or its sialylated form. It was proposed that circulated IgA1 with Tn structures are recognized by autoantibodies specific for GalNac (IgG or IgA), and the formed immune complex is deposited in the kidney, leading to glomerular injury [474]. Autoantibody levels were observed to correlate with disease severity [475]. Aberrant *O*-glycosylation in IgAN was linked to a different expression of two glycosyltransferases, namely, decreased C1GalT1 and increased ST6GalNAc-II. Interestingly, it was observed that biosynthesis of Tn proceeds in the Golgi apparatus instead of in the ER [476].

### 6.3. Antiglycan Antibodies

**Figure 22:**
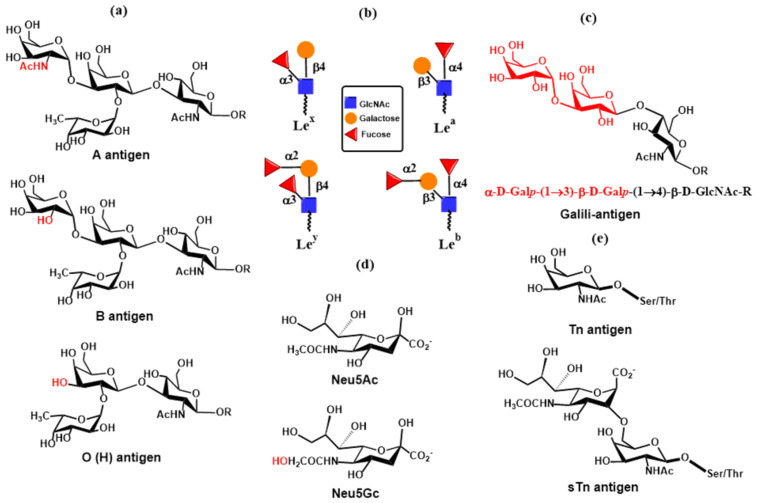
Selected glycan epitopes recognized by human antibodies. Schematic representations structures of (**a**) ABO group antigen; (**b**) Galili antigen; (**c**) sialic acids, where Neu5Ac and Neu5Gc differ only by the presence of additional hydroxyl group (colored in red) in Neu5Gc; (**d**) Lewis antigens; (**e**) Tn and sTn antigens associated with cancer.

Glycocalyx covers all human cells, consisting of enormous amounts of glycoconjugates that contain distinct carbohydrate epitopes [7]. While human epitopes comprise 10 monosaccharides, bacterial carbohydrates also contain several unique monosaccharide residues [477]. Therefore, it is unsurprising that antiglycan antigens are widespread among immunoglobulins that recognize various glycan antigens [441,478]. Antiglycan antibodies can bind to foreign monosaccharides, distinguish between types of glycosidic linkage in disaccharides, or identify a subtle change in the monosaccharide structure. Selected examples of glycan epitopes recognized by human antibodies are shown in Figure 22.

The ABO(H) blood group was discovered more than 100 years ago [479] and represent the most studied antigens. They are present in various tissues throughout the body and in body fluids. The A, B, and O phenotypes are oligosaccharides expressed on red cell membranes as glycoconjugates (Figure 22a). During development, as a response to microbes in the gut, infants develop mainly IgM antibodies [480], where A and B blood groups lead to anti-B and anti-A antibodies, respectively, and the O determinant develops both antibodies [481]. In vivo, such antibodies can, in incompatible transfusions, activate the complement process, which can lead to death. Two retaining glycosyltransferases are involved in the biosynthesis of the human ABO(H) blood group antigens. An *N*-acetylgalactosaminyltransferase (GTA) transfers the GalNAc residue from a UDP-GalNAc donor to the H-antigen acceptor via an α-1,3-linkage, forming the A antigen. The B antigen is biosynthesized by galactosyltransferase (GTB), which transfers galactose from a UDP-galactose donor to the H-antigen acceptor via an α-1,3-linkage, forming the B antigen (Figure 22a). The solved crystal structures of GTA and GTB revealed that both GTs differ only in the identity of four critical amino acid residues [482]. Recently, various studies investigated associations between the ABO blood group and COVID-19. The rates of SARS-CoV-2 infection are linked with the O blood type, which provides some protection against disease progression. On the contrary, blood type A is assumed to be a risk factor correlated with the severity and mortality of COVID-19 infection [483].

The Lewis antigens are present in many cell types of various tissues and body fluids [484]. Similarly to the ABO blood group, Lewis antigens are terminal oligosaccharide structures that can be attached to β-Gal presented on *O*- and *N*-linked glycans and glycolipids (Figure 22b). The Lewis antigens are synthesized from type 1 and 2 oligosaccharide precursors by fucosyltransferases. Human α-1,3/4 and α1,2-fucosyltransferases carry out the last two steps of Lewis blood antigen biosynthesis [90]. Modifying the GlcNAc residue on the terminal *N*-acetyllactosamine by adding l-fucose via α-1,3- and α-1,4-linkages leads to Lewis x (Le^x^) and Lewis a (Le^a^) antigens. Then, adding l-fucose via an α-1,2-linkage to the galactose residue on Le^x^ and Le^a^ leads to Lewis y (Le^y^) and Lewis b (Le^b^) antigens, respectively [92]. Anti-Lewis antibodies are usually naturally occurring as IgM.

The α-Gal epitope (Galili epitope, α-d-Galp-(1→3)-β-d-Galp-(1→4)-β-d-GlcNAc-R) contains disaccharide where the α-1,3-linkage joints two galactose residues (Figure 22c) [485]. The biosynthesis of the Galili epitope is carried out by retaining α-1,3-GalT (α-1,3-galactosyltransferase) using uridine-5-diphosphogalactopyranose (UDPGal) as the donor. The terminal α-Gal epitope is a xenoactive antigen associated with organ rejection in xenotransplantation [486]. The α-Gal epitope is produced by non-primate mammals, lemurs, and New World monkeys. However, humans, apes, and Old World monkeys lost α-Gal epitopes a long time ago, and humans produce the natural antibodies IgG, IgM, and IgA within the first months of life [487]. Interestingly, α-Gal immunoglobulin IgE has been associated with an allergic reaction to red meat in connection with tick bites [488].

While *N*-acetylneuraminic acid (sialic acid, Neu5Ac) is widespread in human glycans, the other sialic acid, *N*-glycolylneuraminic acid (Neu5Gc), is not present in humans (Figure 22d) due to an irreversible mutation of the human gene responsible for the production of Neu5Gc a long time ago [489]. Neu5Gc-specific antibodies do not recognize Neu5Ac despite the subtle structural difference between both sialic acids [490].

Aberrant glycosylation is characteristic of cancer cells and various glycan antigens, including enhanced sialyl Lewis structures, β-1,6-*N*-glycan branches, polylactosamine chains, core fucosylation, or Tn and sialylated Tn antigens (Figure 22e), are often associated with cancer progression [9,38,396,398,400]. The truncated *O*-glycan sTn is overexpressed in numerous human adenocarcinomas, including breast, ovarian, bladder, cervical, colon, pancreatic, and lung cancers [491,492,493] and has been a target for the development of monoclonal antibodies (mAbs) for diagnosis and therapeutic purposes for years. In addition, a repertoire of various anti-sTn antibodies have been generated using IgM, IgG, and IgA [441,494]. Some of them have entered clinical trials with generally well-tolerated therapy. However, the results were unsatisfactory, with a short delay of disease progression [494]. New antibodies are continuously produced, and a recent investigation of the recognition of sTn by the L2A5 antibody revealed information at the atomic level that can be useful in rational tuning and potential application of this anti-sTn antibody for diagnosing and treating various cancers [495].

### 6.4. Glycan-Based Vaccines

The history of glycan-based vaccines started more than 100 years ago when the capsular polysaccharide of *Streptococcus pneumoniae* was isolated in 1917 [496]. Thirty years later, the first two vaccines based on capsular polysaccharides (CPS) against *Streptococcus pneumoniae* were approved in the USA [497]. Then, CPS-based vaccines have been developed against *meningococcus*, *pneumococcus*, *Salmonella typhi*, *Neisseria meningitides*, and *Haemophilus influenzae* type b (Hib) and were licensed in the last century [498]. A drawback of this approach was that CPS vaccines were ineffective for children under 2 years of age [499]. Later, glycoconjugate vaccines were introduced with carbohydrate antigens linked to a carrier protein [500,501]. Over the years, vaccines have become an efficient and cost-effective approach to controlling infections caused by pathogens. Consequently, much effort has been focused on developing carbohydrate vaccines against various diseases and bacterial infections, viruses, fungal and parasite infections, and cancer [498,499,502,503]. Recently, tremendous progress has been made in solving challenges inherent to carbohydrate-base vaccines. These included weak (in a millimolar range) carbohydrate–protein non-covalent interactions [5], which are in nature enhanced by multivalent binding [504]; the heterogeneity of glycans that usually appear on the same protein as glycoforms and the antibodies need to adjust heterogeneity requirements; and conformational flexibility of glycans that occur as a dynamic mixture of conformers [5]. Moreover, an anti-glycan response is T cell independent and therefore short lived, less robust, and usually involves IgM. Additionally, since glycans decorate all cells, they may be considered as self by the host immune cells.

The production of glycoconjugate vaccines involves the selection of the antigen source, the carrier, the conjugation method, and the adjuvant. Historically, polysaccharides isolated and purified from biological sources were used as the antigen part. This led to heterogeneous and undefined antigen structures. In the last two decades, many efforts have been focused on synthetic complex oligosaccharides representing minimal antigens to produce better-defined antigens. Various synthetic, chemoenzymatic, and glycoengineering procedures were developed to generate glycan antigens with high purity and well-defined structures. Recently, polyvalent vaccines have been designed to increase protection against pathogens [505]. Various methods are used to conjugate carbohydrate antigens with immunogenic carrier proteins, including tetanus toxoid (TT), diphtheria toxoid (DT), Cross-Reactive-Material-197 (CRM_197_), or the outer membrane protein complex of meningococcus B (OMPC). In addition, various adjuvants have been used safely for decades to help produce a strong response to protect the person from the disease [506]. Several immune conjugate versions of vaccines are now used in clinics or are in development. Several reviews describe progress related to the subject [507,508,509,510].

## 7. Concluding Remarks

Glycans decorate all immune cells and bear important bioinformation content decoded by glycan-binding proteins (GBPs), such as selectins, galectins, and Siglecs. GBP–glycan interactions are sensitive to subtle changes in the glycan epitopes and distinguish between the host (self), microbial (non-self), and tumor (altered self) antigens. GBPs thus identify structural changes in the glyco (sweet) code between health and disease stages and provide the molecular basis for many diseases. Targeting these interactions offers therapeutic potential. Recent progress in understanding GBP–glycan interactions accelerated the development of glycan-targeted therapeutics using different strategies, including the modulation of GBP–glycan interactions, GBP expression, alterations in the biosynthesis of glycans, immunotherapy, anti-glycan mAbs and vaccines, and cleaving glycan epitopes. Many diverse molecule antagonists, mAbs, and vaccines were developed, and despite considerable effort, only a few have reached clinics.

The inhibition of GBP–glycan interactions is the most commonly used approach to develop glycan-targeted therapeutics. Numerous diverse glycomimetic, non-carbohydrate, and polysaccharide agents inhibiting these interactions have been developed. However, only a few compounds have shown promising results in clinical trials. Several obstacles hampered progress in the search for GBP–glycan inhibitors. The GBP–glycan interactions are generally of low affinity, and the observed high affinity in biological systems is often attributed to multivalency. Indeed, micro-domains or clusters formed by GBPs lead to tight binding with their glycan ligands. Additionally, the pharmacokinetic properties of glycomimetics are usually insufficient for a therapeutic application, and the production of antibodies is often laborious and expensive. In selectins, disclosing sLe^x^ as the minimal binding determinant led to the developing of its glycomimetic Cylexin (Cytel Corp.). However, insufficient results in a phase II trial led to the program’s cancellation. Later, Bimosiamose (Texas Pharmaceuticals), Rivipansel (GlycoMimetics), and Uproleselan (Glycomimetics) were designed, but only Uproleselan is still in development. Also, various carbohydrate-based galectin inhibitors, including chemically modified derivatives of galactose, talose, lactose, *N*-acetyllactosamine, and pectin, were synthesized and tested in pre-clinical and clinical trials. Some of these trials’ results remain unknown, while some have been withdrawn or terminated, such as Belapectin (Galectin Therapeutics) and Davatan (Galectin Therapeutics). Progress in understanding the key structural features required for the specificity and potency of inhibitors will facilitate the design of novel, specific, and potent inhibitors.

Monoclonal antibodies targeting glycans are essential in human health and basic and translational research. In basic research, they are used to identify glycans and investigate their expression and biological roles. In the clinic, anti-glycan mAbs are used to diagnose and monitor disease progression. Anti-glycan mAbs in clinics distinguish between glycans in healthy organisms and those in cancer cells, bacteria, viruses, fungi, or parasites. Therefore, many antibodies that recognize various TACAs and pathogen glycans have been tested in numerous clinical trials. Recently, the FDA approved Dinutuximab/Unituxin (National Cancer Institute) and Naxitamab/Danyelza (Memorial Sloan Kettering Cancer Center) to target ganglioside GD2 for cancer treatment. However, producing anti-glycan mAbs is laborious and expensive. The proper glycosylation that affects biological efficacy is especially critical for the quality of mAbs. Therefore, high-quality anti-glycan mAbs are available only for a small fraction of carbohydrate determinants, and it is clear that there is a need for new technologies for anti-glycan antibody development.

Despite challenges associated with low immunogenicity glycans and the production of antibodies, various carbohydrate-based vaccines against pathogens and cancer have been developed, and some are widely used. For example, carbohydrate-based vaccines in humans against the bacteria *Haemophilus influenzae* type b (Pentacel from Sanofi Pasteur, Hiberix, Merck and Co.), *S. pneumoniae* (PneumoVax, Merck and Co.; Prevnar 13, Pfizer), *Neisseria meningitidis* (MenACWY vaccines, Menveo and MenQuadfi; MenABCWY vaccine, Penbraya), and dengue virus (Dengvaxia, Sanofi-Pasteur) have been licensed by the FDA. A vaccine against malaria disease (PfSPZ, Sanaria) is in phase IV clinical trial. Aberrant glycosylation leads to the generation of TACAs used to develop anti-cancer vaccines. Despite considerable efforts and many vaccines targeting glycans in pre-clinical and clinical trials, only some vaccines stimulate a sufficient immune response. For example, Theratope (Biomira), GM2-KLH (Memorial Sloan Kettering Cancer Center), OPT-822 (OBI Pharma), and Racotumomab (Molecular Immunology Center in Havana) targeting sTn antigen, ganglioside GM2, glycolipid Globo H, and ganglioside GM3, respectively, surpassed phase III trials. Until now, no anti-cancer vaccine targeting glycans has been approved by the FDA. Recent progress in bioinformatics, structural characterization of glycans in different types of cancer, antibody isolation techniques, and high-throughput glycan microarray methodologies will provide a better understanding of the nature of the GBP–glycan interactions, which could lead to the rational design of improved anti-glycan vaccines.

Recently, immune checkpoint inhibitor (ICI) therapy was developed to reshape the tumor microenvironment. Several ICSs targeting the PD-1/PD-L1 signaling pathway, such as pembrolizumab (Keytruda), nivolumab (Opdivo), and cemiplimab (Libtayo), have been approved by the FDA. However, there is a lack of a predictive biomarker for patient selection, and their success remains limited, with only a subset of patients achieving sustained responses. Progress in understanding the ICI mechanism of action and tumor pathophysiology in specific tumor types will likely increase the efficiency of the ICI treatment.

Glycan–GBP interactions are central axes of multiple aspects of cancer, and another strategy for interfering with these interactions is modification of the glycan structure (glyco-code). This modification can be obtained by influencing genes coding GT, the use of inhibitors of glycan biosynthesis, or specific antibodies. In recent years, altering the biosynthesis of carbohydrate determinants with carbohydrate processing inhibitors (CPIs) has emerged as an interesting approach. CPIs disturb glycosylation pathways by inhibiting GT involved in biosynthesis. The result is altered glycan structures, which GBP does not recognize. Different strategies have been used to develop GT inhibitors. A natural approach was to design mimetics of the sugar nucleotide donor, acceptor, and bisubstrate mimetic combining features of the donor and acceptor. Another strategy is based on progress in understanding the catalytic mechanism and determining the transition state structure of the enzymatic reactions of GTs. It is well known that the best inhibitors of an enzymatic reaction are transition state analogs (TSAs). Therefore, considerable effort has been focused on the development of TSA mimetics. Maybe the most successful story is the development of two drugs, zanamivir and oseltamivir, inhibitors of sialidase that play a crucial role in the survival of the influenza virus. These compounds are transition state analogs of the catalytic reaction of a viral enzyme, sialidase, and belong to the so-called carbohydrate processing inhibitors (CPIs). The design of CPIs is in its infancy and requires a combination of glycobiology, medicinal chemistry, and molecular modeling to guide the rational design of such agents. Though developing CPIs is challenging due to the specific features of the catalytic reaction of GTs, they represent a promising route for discovering new therapeutic agents.

The development of new therapeutic agents using the above strategies has some limitations. Advances in glycomics and glycoproteomics have furnished recent progress in detecting altered glycosylation, characterization of glycan structural properties, deciphering the catalytic mechanism of GTs, and understanding glycan functions in pathological processes. Despite the challenges associated with the complexity of GBP–glycan interactions, these findings provide the groundwork for continually developing various diverse and innovative approaches that will lead to specific and potent glycan-targeted therapeutics.

## Data Availability

No new data were created or analyzed in this study. Data sharing is not applicable to this article.
